# A century of vitamin E research: The innovative journey from basic biology to synthetic bio‐manufacturing

**DOI:** 10.1111/jipb.70235

**Published:** 2026-03-22

**Authors:** Ruiqi Zhang, Yuqing Ren, Yuehe Zhao, Hongyan Zheng, Yanzhong Luo, Yuan Liu, Lei Wang, Lan Zhang

**Affiliations:** ^1^ Biotechnology Research Institute Chinese Academy of Agricultural Sciences Beijing 100081 China; ^2^ National Nanfan Research Institute (Sanya) Chinese Academy of Agricultural Sciences Sanya 572024 China

**Keywords:** antioxidant, biosynthesis, metabolic engineering, vitamin E

## Abstract

Since its discovery in 1922, vitamin E research has evolved from the search for a mysterious “reproductive factor” to the exploration of a diverse family of bioactive molecules central to plant physiology and human health. This review traces a century of progress, highlighting advances in our understanding of vitamin E's chemical composition, antioxidant and non‐antioxidant functions, biosynthetic pathways, and intricate regulatory networks in plants. Recent breakthroughs, such as the discovery of the seed‐specific esterase, VTE7, revealed a direct phytol‐recycling route linking chlorophyll degradation to tocopherol synthesis. This discovery has opened new possibilities for metabolic engineering. To overcome the persistent bottlenecks of low natural abundance and costly extraction, we also examine two production strategies: chemical synthesis and biotechnological synthesis. While chemical routes remain dominant, they yield racemic mixtures with reduced bioactivity. Emerging synthetic biology approaches, including microbial platforms capable of producing natural vitamin E configurations from key precursors, such as farnesene, mark a new paradigm for green and efficient manufacturing. Looking ahead, future directions include the intelligent evolution of catalytic enzymes, elucidation of transmembrane precursor transport, and exploration of rare homologs such as tocomonoenols. Together, these innovations promise to redefine the molecular and industrial landscape of vitamin E research for the next century.

## Graphical abstract

For a century, Vitamin E has been studied for its health benefits. This review traces its journey from basic biological discovery, and the recent identification of its link to chlorophyll degradation, to the modern era of sustainable microbial manufacturing, highlighting the innovative technologies that define its future production.

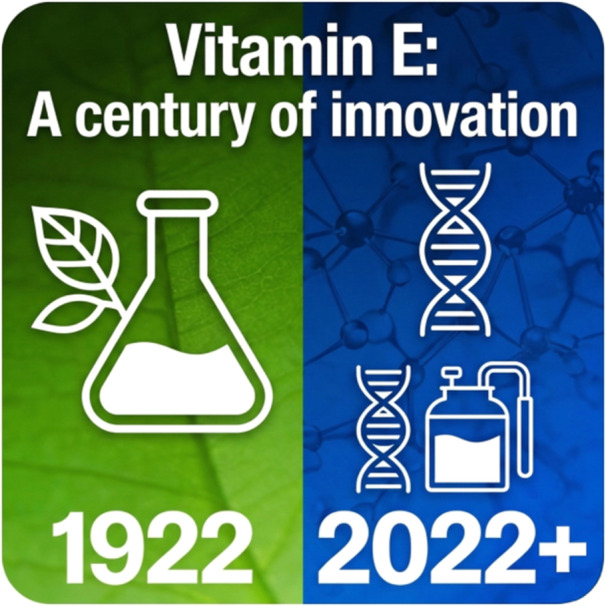

## INTRODUCTION

**Figure 1:**
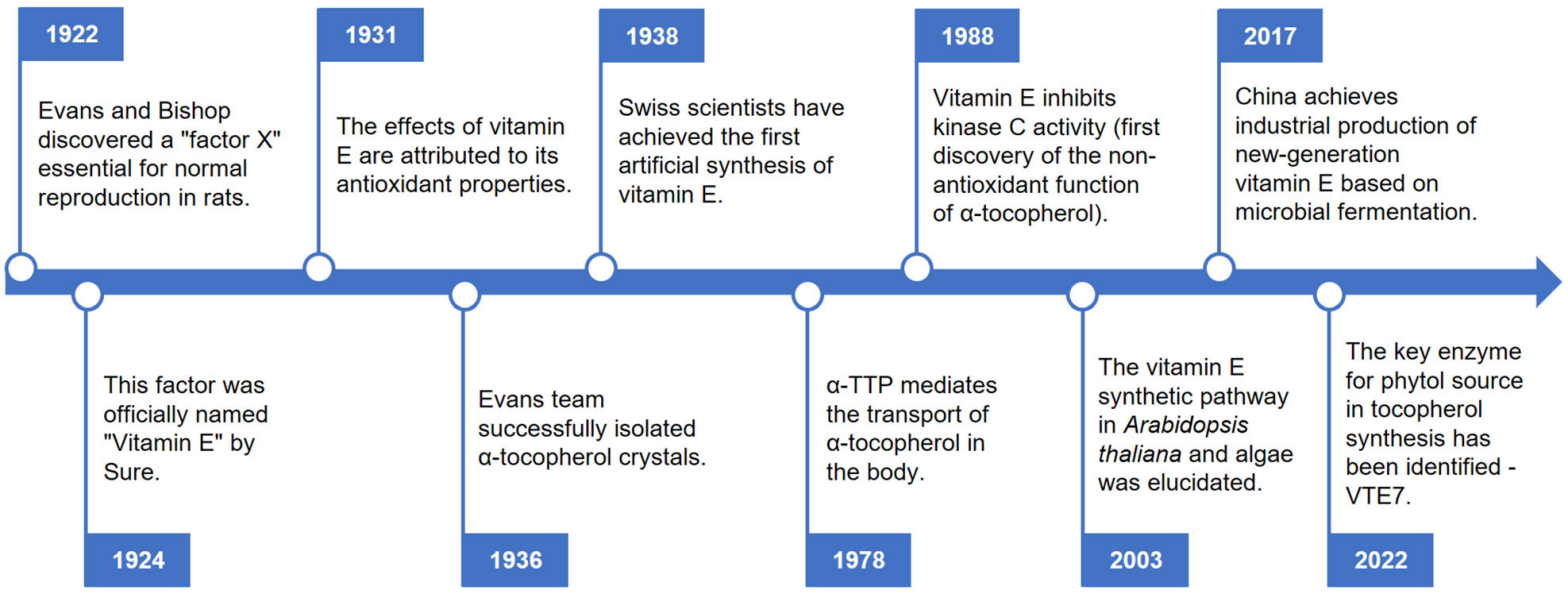
Key milestones in the century of vitamin E research 1922—Evans and Bishop identify a lipid‐soluble factor essential for reproduction; 1924—Sure names it “vitamin E”; 1931—Olcott and Mattill attribute its action to antioxidant properties; 1936—Evans' team isolates crystalline α‐tocopherol; 1938—Karrer achieves the first chemical synthesis; 1975—Discovery of α‐TTP (α‐Tocopherol transfer protein)‐mediated transport; 1988—Identification of α‐tocopherol inhibition of protein kinase C (first non‐antioxidant function); 2003—Pathway elucidation in Arabidopsis and algae; 2017—Microbial fermentation enables industrial‐scale vitamin E production in China; 2022—Discovery of VTE7, revealing direct phytol‐recycling.

A century has passed since Evans and Bishop discovered vitamin E in 1922 (Evans and Bishop, 1922), marking the beginning of one of the most enduring scientific stories in nutrition and biology (Figure 1). What was once a mysterious “micronutrient” has since evolved into a diverse family of bioactive compounds, including tocopherols, tocotrienols, tocomonoenols, and plastochromanol‐8 (PC‐8), with functions extending far beyond classical nutrition. Over the past 100 years, vitamin E research has expanded from biochemistry and medicine into plant physiology, molecular biology, agricultural science, and synthetic biology, reflecting its central role at the interface of health, metabolism, and innovation (Evans and Bishop, 1922; Sure, 1924; Olcott and Mattill, 1931; Karrer et al., 1938; Catignani, 1975; Mahoney and Azzi, 1988; Sattler et al., 2003; Ye et al., 2022).

Our understanding of vitamin E's function has undergone a profound transformation. It was first recognized primarily for its antioxidant activity, as it scavenges free radicals and prevents oxidative damage to biological membranes, forming the molecular foundation for many of its physiological roles (Wagner et al., 1994; Zanetti and Catalá, 2000). Yet decades of research have revealed that its influence extends far beyond antioxidant chemistry. Increasing evidence shows that vitamin E participates in cell‐signaling cascades, regulates gene expression, modulates inflammation, and contributes to diverse metabolic and developmental processes (Buettner, 1993; Azzi, 2018; Kim and Han, 2019; Zingg, 2019). This duality, antioxidant and regulatory, defines vitamin E as both a molecular shield and a signaling integrator.

Because humans cannot synthesize vitamin E *de novo*, its supply depends entirely on dietary intake, making photosynthetic organisms, particularly higher plants, the ultimate natural source (Falk and Munné‐Bosch, 2010). Within plants, vitamin E distribution shows marked tissue and species specificity: Tocopherols predominate in green tissues, whereas tocotrienols with stronger antioxidant potential accumulate mainly in the seeds of certain monocots (Horvath et al., 2006; Falk and Munné‐Bosch, 2010). However, absolute levels remain extremely low, typically in the microgram‐per‐gram fresh‐weight range, and production is constrained by cultivation cost, climate variability, and complex extraction and purification steps (Li et al., 2011). This intrinsic limitation has motivated biofortification strategies, combining metabolic engineering with breeding, to generate high‐vitamin‐E crop germplasm as a sustainable solution for global demand.

Deciphering the biosynthetic pathway of vitamin E has been central to understanding its biological regulation. In plants, algae, and cyanobacteria, the pathway comprises plastidial and cytosolic steps linking the shikimate and methylerythritol phosphate (MEP) routes. While most core reactions have been elucidated, key regulatory enzymes such as homogentisate phytyltransferase (HPT), homogentisate geranylgeranyltransferase (HGGT), MPBQ methyltransferase (MT), and tocopherol methyltransferase (TMT), which determine both isomer composition and total yield, remain active targets for cloning and characterization (Seo et al., 2011; Zeng et al., 2020). The recent discovery of VTE7, an esterase that directly channels phytol from chlorophyll degradation into tocopherol biosynthesis, revealed a new and efficient phytol‐recycling route (Albert et al., 2022). This finding not only establishes a direct link between chlorophyll catabolism and vitamin E biosynthesis but also identifies new targets for metabolic engineering. Nevertheless, major gaps persist in our understanding of rate‐limiting control, precursor trafficking between subcellular compartments, and multilayered transcriptional and post‐translational mechanisms that now define the frontier of vitamin E biology.

In parallel, chemical synthesis, first achieved in 1938, has long dominated industrial production, yielding racemic α‐tocopherol that differs from natural stereoisomers in bioactivity. A century after its discovery, revisiting vitamin E's research trajectory is both timely and instructive. This review synthesizes advances in its discovery, chemical composition, and physicochemical properties; dissects progress in natural and chemical biosynthetic routes; and summarizes its physiological mechanisms, spanning antioxidant to non‐antioxidant functions. While foundational reviews have extensively covered vitamin E biosynthesis in crop biofortification strategies and the evolution of vitamin E production (DellaPenna, 2005; Li et al., 2025; Mène‐Saffrané, 2017; Yang et al., 2024), fewer studies have bridged the gap between basic plant biology and emerging industrial manufacturing paradigms. This review distinguishes itself by integrating a century of biological discovery with the latest technological breakthroughs. Beyond updating the classical biosynthetic pathways with the recently discovered VTE7‐mediated phytol recycling route, we critically evaluate the shift from traditional chemical synthesis to bio‐hybrid approaches and microbial cell factories. Furthermore, we explore frontier technologies such as AI‐driven intelligent enzyme evolution, which promises to revolutionize biocatalyst efficiency. By connecting molecular mechanisms to industrial applications, we aim to provide a comprehensive roadmap for the next century of vitamin E innovation.

## VITAMIN E DEFICIENCY

Deficiency of vitamin E triggers a cascade of interrelated pathophysiological changes, underscoring its central role in maintaining systemic health. Although overt deficiency is rare in individuals with balanced nutrition, the risk rises sharply under certain genetic, metabolic, or acquired conditions (Ouahchi et al., 1995; Cavalier et al., 1998; Di Donato et al., 2010). Hereditary causes include mutations in the α‐TTP gene, leading to ataxia with vitamin E deficiency (Sokol, 1988), and disorders of lipoprotein metabolism such as abetalipoproteinemia (Wetterau et al., 1992) and hypobetalipoproteinemia (Ross et al., 1988). Acquired factors involve fat‐malabsorption syndromes, severe malnutrition, and increased oxidative load from rancid dietary fats (Pignitter et al., 2014). Prolonged low‐fat diets, excessive consumption of highly processed micronutrient‐poor food (Falcão et al., 2019), and certain iatrogenic interventions, including bariatric surgery and lipase‐inhibiting medications, also predispose to deficiency (Melia et al., 1996; McDuffie et al., 2002; Sherf‐Dagan et al., 2021).

The core pathophysiological mechanism involves the loss of chain‐breaking antioxidant protection. Without adequate vitamin E, unchecked lipid peroxidation amplifies oxidative stress, damaging membranes rich in polyunsaturated fatty acids (PUFAs), particularly neuronal myelin, erythrocyte membranes, and retinal photoreceptors (Head and Traber, 2021; Traber and Head, 2021). Neurological symptoms are the most prominent features of vitamin E deficiency, including progressive peripheral neuropathy, which initially presents as sensory impairment (Sokol et al., 1988; El Euch‐Fayache et al., 2014), and spinocerebellar ataxia, regarded as its clinical hallmark (Ben Hamida et al., 1993). Additional manifestations include loss of proprioception and skeletal myopathy. Hemolytic anemia and retinal degeneration, such as pigmentary retinopathy (Yokota et al., 1997, 2000) and macular degeneration (Iwasa et al., 2014), along with impaired immune responses, are also commonly observed. Therapeutic management requires prompt and sustained supplementation, often lifelong. Genetic disorders demand high‐dose α‐tocopherol therapy, whereas patients with malabsorption benefit from water‐soluble derivatives such as tocopherol polyethylene glycol succinate (TPGS) to enhance bioavailability (Traber et al., 1986, 1988). Such interventions effectively raise serum tocopherol concentrations (Sokol et al., 1988), ameliorate neurological symptoms, and arrest disease progression (Sokol et al., 1993).

## MOLECULAR MECHANISMS OF VITAMIN E

### Antioxidant mechanisms

#### Scavenging radicals with vitamin E

Vitamin E is a principal lipophilic antioxidant (Buettner, 1993) that protects PUFAs, particularly the highly oxidation‐sensitive docosahexaenoic acid (DHA), from oxidative damage (Wagner et al., 1994; Zanetti and Catalá, 2000). Free radicals, defined as atoms or molecules containing one or more unpaired electrons, can initiate oxidation of lipids, proteins, nucleic acids, and carbohydrates, thereby compromising cellular integrity. The capacity of vitamin E to quench these radicals forms the molecular basis of its antioxidant function (Gulcin, 2020). Biological systems harbor a diverse spectrum of radical species, including superoxide radical (O_2_•^−^), hydroxyl radical (HO•), alkoxy (RO•), peroxyl (ROO•), aryloxy (ArO•), nitric oxide (•NO), nitrogen dioxide (NO_2_•), thiol (RS•), sulfonyl (RSO_2_•), and carbon‐centered radicals (R•) (Wang et al., 2024b). These radicals vary markedly in reactivity: Relatively weak oxidants such as superoxide and nitric oxide rarely cause direct molecular damage, whereas highly reactive radicals attack biomolecules via hydrogen abstraction, double‐bond addition, or electron transfer, triggering oxidative chain reactions and cellular damage.

Vitamin E encompasses several naturally occurring isomers as well as synthetic stereoisomers, including all‐rac‐α‐tocopherol, each exhibiting distinct radical‐scavenging efficiencies (Sharma et al., 2012; Niki, 2014; Dumanović et al., 2020). The reactivity of tocopherol homologs toward oxygen‐derived radicals follows the order α > β ≈ γ > δ (Burton and Ingold, 1981; Niki et al., 1986; Mukai et al., 1988; Pryor et al., 1988). The phenolic hydroxyl group at the 6‐position of the chromanol ring constitutes the principal site of antioxidant activity, whereas variation in the side chain at the 2‐position has little effect on intrinsic radical‐scavenging reactivity (Kaiser et al., 1990). Consequently, tocopherols and their unsaturated counterparts, tocotrienols, exhibit comparable hydrogen‐donating capacity. However, the presence of three additional *trans*‐double bonds in the tocotrienol side chain imparts greater conformational flexibility, facilitating deeper penetration into lipid bilayers and more effective interaction with lipid radicals. This structural advantage may account for the enhanced antioxidant efficacy of tocotrienols observed in certain systems, in some cases exceeding that of tocopherols (Suzuki et al., 1993; Babura et al., 2017). An intriguing evolutionary question arises as to why tocotrienols, despite their high antioxidant potential, are synthesized by only a limited number of monocot species rather than being widespread across the plant kingdom. One plausible explanation is that tocotrienol accumulation represents an adaptive strategy evolved under specific environmental pressures, such as high light intensity or elevated temperature, albeit at increased metabolic cost. Tocotrienol biosynthesis requires geranylgeranyl pyrophosphate (GGPP) as a precursor, whereas tocopherol synthesis uses phytyl diphosphate (PDP). The formation of GGPP introduces three additional double bonds, necessitating greater reducing power and ATP investment. Thus, tocotrienol synthesis embodies a “high‐input, high‐return” strategy, energetically demanding but conferring enhanced oxidative protection, likely favored only under selective ecological conditions, thereby explaining its restricted distribution among plant species.

**Figure 2:**
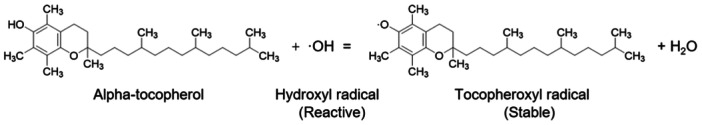
Radical‐quenching mechanism of vitamin E Vitamin E neutralizes reactive radicals through hydrogen atom donation from its phenolic hydroxyl group, forming a relatively stable tocopheroxyl radical and thereby terminating lipid oxidation chain reactions.

The radical‐quenching mechanism of vitamin E operates primarily through hydrogen atom donation, yielding a stable tocopheroxyl radical and a reduced lipid product (Figure 2):

X•+Vit E−OH→XH+Vit E−O•

The resulting tocopheroxyl radical can subsequently react with another radical to form a non‐reactive product or be reduced back to its active form by cellular reductants such as ascorbate, glutathione (GSH), or ubiquinone, thereby sustaining antioxidant capacity (Liebler et al., 1996; Yamauchi, 2007). Through this dynamic cycle of oxidation and reduction, vitamin E serves as a frontline defender of lipid integrity and redox homeostasis in biological membranes.

#### Scavenging nonradical oxidants

Oxidative stress in biological systems is not mediated solely by free radicals; a variety of nonradical oxidants can also induce significant oxidative damage. Among these, hydrogen peroxide (H_2_O_2_), singlet oxygen (^1^O_2_), ozone (O_3_), and hypochlorous acid (HOCl) are particularly important. Although these species lack unpaired electrons, they possess strong electrophilic or oxidizing potential and can readily react with biomolecules, causing structural and functional impairments comparable to those induced by free radicals (Wang et al., 2024b).

During radical‐mediated oxidation, self‐propagating chain reactions are a defining feature, with lipid peroxidation posing one of the greatest threats to biological integrity. This multistage process unfolds as follows. In the initiation phase, highly reactive radicals such as hydroxyl radicals (•OH) attack PUFAs (LH, e.g., arachidonic acid), abstracting a hydrogen atom to form a lipid radical (L•). In the propagation phase, L• rapidly reacts with molecular oxygen to generate a lipid peroxyl radical (LOO•), which subsequently abstracts a hydrogen atom from a neighboring lipid molecule to produce lipid hydroperoxide (LOOH) while regenerating L•, thereby perpetuating the chain reaction:

$$L•\;+\;O_{2}\to \text{LOO}•$$

LOO•+LH→LOOH+L•

Termination occurs when two radicals combine to form a nonradical product, thereby halting the chain reaction, as illustrated by the following reactions:

$$\text{LOO}•\;+\;\text{LOO}•\;\to \;\text{LOOL}+O_{2}$$

LOO•+VitE•→stableproduct

The LOOH generated during propagation is chemically unstable. In the presence of transition metal ions such as Fe^2+^, LOOH can undergo homolytic cleavage, producing highly reactive alkoxy (LO•) and peroxyl (LOO•) radicals that further exacerbate oxidative injury. Subsequent decomposition of LOOH yields reactive aldehydes, including malondialdehyde (MDA) and 4‐hydroxynonenal (HNE), which readily form covalent adducts or crosslinks with proteins and nucleic acids, thereby amplifying cytotoxicity. The consequences of lipid peroxidation extend well beyond localized membrane damage. Oxidative degradation compromises the integrity, fluidity, and permeability of phospholipid bilayers, impairs membrane‐protein function, and, through the action of secondary reactive products, initiates widespread biochemical dysfunction (Chandimali et al., 2025). By intercepting peroxyl and alkoxy radicals and stabilizing LOOHs, vitamin E acts as the principal chain‐breaking antioxidant, preventing the self‐propagating cascade of lipid oxidation and preserving cellular membrane homeostasis.

#### Synergism with other antioxidants

**Figure 3:**
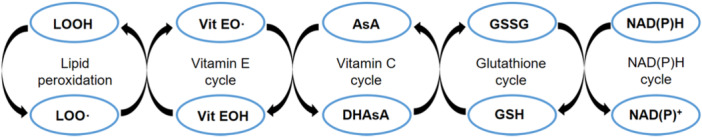
Antioxidant network centered on vitamin E Vitamin E functions as a key component of the cellular antioxidant defense system, acting synergistically with ascorbate (AsA), glutathione (GSH), and NAD(P)H to regenerate its reduced form and maintain cellular redox homeostasis (Blaner et al., 2021).

Vitamin E functions as an integral component of the cellular antioxidant defense network, operating in close cooperation with vitamin C, GSH, and the reducing power of nicotinamide adenine dinucleotide phosphate (NADPH) (Niki and Noguchi, 2004; Cadenas et al., 2016). This interconnected system maintains redox homeostasis through a sequential regeneration cycle. When vitamin E scavenges lipid peroxyl radicals within membranes, it is oxidized to a tocopheroxyl radical. This radical is subsequently reduced back to α‐tocopherol by ascorbate (vitamin C), which itself is converted into an ascorbyl radical. The ascorbyl radical is then recycled through the action of GSH, which donates electrons and is oxidized to GSH disulfide (GSSG). Finally, GSH reductase, using NADPH as an essential electron donor, reduces GSSG back to GSH, thereby completing the antioxidant cycle (Figure 3; Cadenas et al., 2016). Through this finely tuned redox cascade, vitamin E is continuously regenerated and maintained in its active form, ensuring sustained protection of membrane lipids against oxidative stress. This dynamic interplay highlights that the antioxidant efficacy of vitamin E is not an isolated property but rather depends on the coordinated function of the broad cellular antioxidant network.

The interconnection between vitamin E and phospholipid hydroperoxide metabolism is particularly evident within the GSH‐GPX4 antioxidant axis. Selenium‐dependent GSH peroxidase 4 (GPX4) specifically reduces phospholipid hydroperoxides, thereby shielding biological membranes from peroxidative damage in a process critically dependent on GSH availability (Ursini et al., 1982). Together, vitamin E and the GSH‐GPX4 system play an essential role in preventing ferroptosis by cooperatively eliminating LOOH and blocking the iron‐driven re‐initiation of lipid‐radical chain reactions, thus suppressing phospholipid peroxidation and programmed cell death (Carlson et al., 2016; Jiang et al., 2021). This protective mechanism further relies on the combined action of GSH, GPX4, and dietary selenium to ensure efficient LOOH detoxification and to prevent the propagation of iron‐catalyzed radicals (Carlson et al., 2016; Ingold et al., 2018). Collectively, these findings indicate that high‐dose vitamin E supplementation alone is unlikely to confer maximal protection. Instead, a synergistic reinforcement strategy ensures adequate levels of vitamin C, cysteine as a precursor for GSH biosynthesis, and selenium to maximize the efficacy of the intracellular antioxidant network. From a translational perspective, developing biofortified crops enriched not only in vitamin E but also with enhanced cysteine and selenium metabolism represents a promising direction for the next generation of functional foods.

### Absorption and transport of vitamin E

As a fat‐soluble compound, vitamin E absorption is tightly coupled to dietary lipid digestion and occurs through a multistage physiological process. In the human intestine, vitamin E is incorporated into mixed micelles together with dietary fats and bile salts and is absorbed across the brush‐border membrane of enterocytes, primarily via passive diffusion (Gallo‐Torres, 1970). Within intestinal mucosal cells, triglycerides, phospholipids, cholesterol, vitamin E, and apolipoproteins are reassembled into chylomicrons (Bjørneboe et al., 1990). These chylomicrons are transiently stored as secretory particles and subsequently released into the lymphatic circulation via exocytosis, ultimately entering the bloodstream (Bjornson et al., 1976; Rigotti, 2007). In plasma, lipoproteins serve as the principal carriers of lipid‐soluble antioxidants, including vitamin E. Circulating α‐tocopherol concentrations correlate closely with total plasma lipid levels. Fractional distribution studies in humans reveal that tocopherols are transported predominantly by low‐density lipoproteins (LDL) and high‐density lipoproteins (HDL) in approximately equal proportions, whereas very‐low‐density lipoproteins (VLDL) and other lipoprotein fractions together account for less than 20% of the total vitamin E pool (Perugini et al., 2000).

### Distribution and metabolism of vitamin E

The liver exhibits a remarkable capacity to discriminate among vitamin E isoforms, a property that fundamentally determines their systemic distribution and biological activity. Following dietary intake, most vitamin E is incorporated into chylomicrons, which deliver it to the liver parenchymal cells via remnant lipoprotein transport (Bjørneboe et al., 1987). Within hepatocytes, α‐TTP, a small cytosolic liver protein with distinct affinities for individual tocopherol and tocotrienol forms, mediates the selective recognition and mobilization of α‐tocopherol for secretion into the circulation (Kaempf‐Rotzoll et al., 2003; Stocker, 2004). Through this process, α‐TTP preferentially incorporates α‐tocopherol into plasma lipoproteins, including VLDL, HDL, and LDL, thereby maintaining relatively stable α‐tocopherol concentrations in blood and peripheral tissues (Arita et al., 1995; Takada and Suzuki, 2010). In contrast, other vitamin E isoforms, such as γ‐tocopherol, are preferentially retained and metabolized in the liver. This selective handling explains why α‐tocopherol constitutes approximately 90% of circulating vitamin E, despite γ‐tocopherol accounting for about 70% of dietary vitamin E intake (Traber, 1999). Until recently, tocopherol‐binding proteins had not been identified in plants. However, Bermúdez et al. (2018) reported the discovery of a tomato chloroplast‐targeted protein, SlTBP, capable of binding α‐tocopherol. Knockdown of *SlTBP* resulted in reduced tocopherol accumulation in both leaves and fruits, suggesting a potential role in vitamin E trafficking within plant tissues, though direct *in vivo* evidence for its transport function remains to be established.

Vitamin E can also undergo spontaneous intermembrane exchange between lipoproteins and cells; however, receptor‐mediated pathways significantly influence its tissue‐specific delivery and distribution. Members of the LDL receptor superfamily (Traber and Kayden, 1984; Thellman and Shireman, 1985) and scavenger receptor class B type I (SR‐BI) (Goti et al., 1998; Rigotti et al., 2003) are thought to regulate the directionality, rate, and organ specificity of α‐tocopherol uptake. Structurally, vitamin E anchors its hydrophobic phytyl tail within the phospholipid bilayer, functioning in a manner analogous to cholesterol but exhibiting distinct kinetic behavior (Taylor et al., 2025). Tocotrienols differ from tocopherols by the presence of three *trans* double bonds in the side chain, which impart a bent and more flexible conformation. This enhanced conformational mobility facilitates deeper penetration into lipid membranes and stronger interactions with unsaturated lipids, thereby enhancing antioxidant activity (Tan, 2005; Bardhan et al., 2011). Molecular modeling studies suggest that the unsaturated tocotrienol tail undergoes dynamic movement within the membrane, resembling a pendulum, in contrast to the relatively rigid and linear side chain of tocopherols. This structural flexibility likely contributes to the superior bioactivity of tocotrienols (Bardhan et al., 2011). Excess α‐tocopherol and non‐α forms are catabolized into carboxyethyl‐hydroxychroman (CEHC) derivatives. The initial oxidative step is catalyzed by cytochrome P450 enzymes, such as CYP3A4 and CYP4F2, which hydroxylate the terminal end of the phytyl chain (ω‐hydroxylation), enabling subsequent β‐oxidation to produce α‐CEHC. These metabolites are then conjugated with glucuronic acid or sulfate to enhance solubility and are ultimately excreted via urine or bile (Brigelius‐Flohé and Traber, 1999; Kiyose et al., 2001; Mustacich et al., 2006).

### Gene expression regulation

Over the past two decades, extensive evidence has demonstrated that vitamin E availability modulates gene expression across multiple tissues and species (Azzi, 2018; Kim and Han, 2019; Zingg, 2019). These studies generally fall into two main categories. The first explores global expression profiles under varying concentrations or exposure durations of vitamin E, comparing tissues or cultured cells subjected to deficient, adequate, or enriched conditions. Most such investigations have employed vitamin‐E‐deficient or ‐supplemented diets in mice or rats to examine transcriptional responses in organs, including the liver, lung, skeletal muscle, cerebral cortex, and testes (Gohil et al., 2003; Barella et al., 2004; Rota et al., 2004; Hyland et al., 2006; Nier et al., 2006; Oommen et al., 2007; Mustacich et al., 2009). Differentially expressed genes identified by microarray analysis have subsequently been validated at both the transcript and protein levels using reverse transcription quantitative PCR (RT‐qPCR) and immunoblotting.

The second line of research investigates whether vitamin E directly or indirectly modulates transcriptional regulation by interacting with nuclear factors or intracellular signaling pathways. Although fewer in number, these studies provide compelling evidence that α‐tocopherol and related metabolites can influence gene expression via nuclear receptor‐mediated mechanisms. For instance, α‐tocopherol activates peroxisome proliferator‐activated receptor γ (PPARγ), leading to the upregulation of adipocyte differentiation markers such as adiponectin, while γ‐tocopherol induces the vitamin E‐metabolizing enzyme CYP4F2 through activation of the pregnane X receptor (PXR) (Zingg, 2015, 2019; Kim and Han, 2019). Vitamin E has also been shown to activate nuclear factor erythroid 2‐related factor 2 (NRF2), enhancing the transcription of antioxidant genes, including those involved in glutathione biosynthesis, such as glutathione synthase, and mitigating oxidative injury (Zingg, 2019). In addition to transcriptional activation, vitamin E exerts indirect regulatory effects by suppressing key pro‐inflammatory pathways. It inhibits protein kinase Cα (PKCα), leading to downregulation of nuclear factor‐κB (NF‐κB)‐mediated cytokines such as tumor necrosis factor‐α (TNF‐α) and IL‐6, and suppresses 5‐lipoxygenase activity, reducing the formation of the inflammatory mediator LTB_4_ (Zingg, 2019). Omics‐based analyses further reveal tissue‐specific transcriptional responses to vitamin E status: Chronic vitamin E deficiency downregulates genes involved in glutathione biosynthesis (γ‐GCS, GS) while upregulating the scavenger receptor CD36 in the liver, whereas in T cells, it enhances the expression of cell‐cycle regulators (e.g., *Ccnb2*, *Cdc2*), thereby promoting immune function (Kim and Han, 2019; Zingg, 2019). Together, these studies highlight vitamin E as a multilevel regulator of gene expression, influencing metabolic, antioxidant, and immune pathways through both receptor‐dependent and redox‐mediated mechanisms.

## APPLICATION OF VITAMIN E

Given its broad physiological roles, vitamin E holds vast potential in the pharmaceutical, nutraceutical, cosmetic, food, and animal‐feed industries.

### Pharmaceutical and health applications

In medicine, vitamin E is used both as an essential nutrient and as a therapeutic or adjunct agent in conditions associated with oxidative stress. Its potent antioxidant capacity protects cellular membranes from lipid peroxidation, conferring particular benefits to neurological and cardiovascular health. Pharmaceutical formulations include soft‐gel capsules, emulsions, and tablets, as well as combination products such as calcium‐vitamin E tablets or CoQ10‐vitamin E soft gels (Mohd Zaffarin et al., 2020). Beyond its classical α‐tocopherol form, which is characterized by high bioavailability, increasing attention has been directed toward tocotrienols, which exhibit distinctive biochemical properties and possess potential benefits in metabolic syndrome and cardiovascular diseases (Aggarwal et al., 2010; Ahsan et al., 2014). Clinical and experimental studies suggest that vitamin E supplementation can lower fasting glucose levels, reduce oxidative stress, and enhance vascular integrity, thereby contributing to the prevention of atherosclerotic lesion development (Jayusman et al., 2014; Tang et al., 2014).

### Dermatological and cosmetic uses

In dermatology, vitamin E serves as a protective antioxidant against UV radiation, delaying skin aging by promoting collagen synthesis and preventing collagen degradation (Pedrelli et al., 2012; Kanchi et al., 2017). It is therefore widely incorporated into cosmetic formulations for its moisturizing, anti‐inflammatory, and photoprotective properties.

### Anticancer and preventive roles

Vitamin E and its derivatives also show anticancer potential through multiple mechanisms, including inhibition of tumor cell proliferation, suppression of angiogenesis, modulation of growth factor‐mediated signaling pathways, and induction of apoptosis (Abraham et al., 2019). Collectively, these pleiotropic actions underscore vitamin E's versatility as both an essential nutrient and a bioactive compound, bridging redox biology with therapeutic and preventive innovation.

### Vitamin E in foodstuffs

Vitamin E is a dietary antioxidant naturally present in plant oils, nuts and seeds, vegetables, and fruits. Over the past two decades, growing awareness of the links between oxidative stress, inflammation, and chronic diseases has intensified interest in the vitamin E content of foods. As an essential micronutrient, vitamin E cannot be synthesized endogenously and must be obtained from the diet. According to the U.S. Food and Drug Administration (FDA), the recommended dietary allowance (RDA) for adults and children aged 4 years and older is 15 mg per day (Food and Drug Administration, HHS, 2016). In the food industry, vitamin E serves a dual role. First, as a natural food antioxidant (additive code E306), it is incorporated into edible oils, fried products, baked goods, and meat items to inhibit lipid oxidation, delay rancidity, and extend shelf life. It also offers a clean‐label alternative to synthetic antioxidants such as butylated hydroxytoluene (BHT) and butylated hydroxyanisole (BHA), aligning with consumer demand for natural formulations. Second, vitamin E acts as a nutrient fortifier, added to dairy products, infant formulas, and breakfast cereals to supplement dietary intake and ensure adequate nutrition across populations.

In China, the use of vitamin E in foods is governed by two national standards: The National Food Safety Standard for the Use of Nutritional Fortifiers in Foods (GB 14880‐2012) and the National Food Safety Standard for the Use of Food Additives (GB 2760‐2024). Under GB 14880, vitamin E is permitted as a nutritional fortifier only in designated food categories, each with clearly defined maximum addition levels (Table 1). The allowable concentrations range from 5 mg/kg to 1,450 mg/kg, depending on factors such as food matrix compatibility, target population intake, and processing stability. Fortification is therefore not universally allowed across all food types, reflecting a controlled approach to ensure nutritional efficacy and consumer safety. Under GB 2760, vitamin E is additionally authorized for use as an antioxidant additive in specified food categories, with a general maximum limit of 0.2 g/kg. Collectively, these standards establish a comprehensive regulatory framework governing the dual nutritional and technological functions of vitamin E use in food production, ensuring its safe and appropriate application in food processing and fortification.

**Table 1:** Vitamin E as a nutritional fortifier: Allowable addition levels by food category according to Chinese National Food Safety Standards (GB 14880‐2012)

Food categories	Allowable content (mg/kg)
Gum‐based candy	1,050–1,450
Margarine & similar products	100–180
Vegetable oil	100–180
Formulated milk powder (general)	100–310
Solid beverage	76–180
Formulated milk powder (maternal)	32–156
Soy flour, soy milk powder	30–70
Ready‐to‐eat cereals	50–125
Jelly	10–70
Formulated milk powder (child)	10–60
Beverages	10–40
Soy milk	5–15

### Animal nutrition

Vitamin E serves as an essential liposoluble antioxidant and is critical in animal nutrition and health. Its vital roles include bolstering antioxidant defenses, modulating immune function, enhancing reproductive performance, and improving meat and egg product quality. In recent years, the growing market relevance of vitamin E in animal feed has become increasingly evident. For instance, the global animal‐feed vitamin E additives market was valued at approximately USD 574.4 million in 2024 and is projected to grow at a compound annual growth rate (CAGR) of 4.1% through 2030 (Grand View Research, 2024). In the same year, vitamin E accounted for the largest share of the feed‐vitamins sector, representing 29.2% of total revenue, while the overall feed‐vitamins market is expected to expand at a CAGR of approximately 4.4% through 2030 (Mordor Intelligence, 2024). These contemporary statistics highlight that vitamin E is not merely a micronutrient but remains a cornerstone additive supporting modern livestock production, feed industry growth, and the evolving requirements of intensive animal husbandry systems.

#### As an antioxidant

Vitamin E protects cellular membranes from oxidative injury by scavenging reactive oxygen species (ROS) and interrupting lipid peroxidation reactions involving PUFAs (Azzi and Stocker, 2000; Traber, 2007). Numerous studies have demonstrated that dietary vitamin E enrichment enhances the oxidative stability of animal tissues (Galobart et al., 2001; Cortinas et al., 2005; Villaverde et al., 2008). Using a “breeder‐semen‐embryo” model, Surai et al. (2019) showed that dietary supplementation with vitamin E markedly increased α‐tocopherol concentrations in reproductive tissues, by approximately twofold in semen and 13‐fold in chick liver, and activated the antioxidant enzyme network, thereby forming a central axis of antioxidant defense in poultry. This mechanism effectively mitigates oxidative stress caused by high‐PUFA diets and thermal stress, underscoring vitamin E's role as a frontline cellular protector.

#### Effects on immunity and inflammation

The functionality of immune cells depends strongly on the integrity of their membranes. During immune responses, macrophages and neutrophils generate ROS through a “respiratory burst” to destroy pathogens; however, excessive ROS can damage immune cells themselves, leading to reduced functionality or apoptosis. As a major lipid‐soluble antioxidant, vitamin E preferentially integrates into immune‐cell membranes, neutralizing excess radicals and preserving membrane structure and function, thereby sustaining immune responsiveness (Lewis et al., 2019). Inflammation is a vital aspect of immune defense, but chronic or excessive inflammation can compromise growth, performance, and overall health. Vitamin E modulates inflammatory signaling by reducing pro‐inflammatory mediators such as prostaglandin E_2_ (PGE_2_), interferon‐γ (IFN‐γ), and TNF‐α, while upregulating the anti‐inflammatory cytokine transforming growth factor‐β1 (TGF‐β1), which supports T‐cell differentiation and immune balance (Moriguchi et al., 1993; Lee and Han, 2018). Supplementation enhances lymphocyte proliferation, antibody production, natural killer cell activity, and cytokine secretion (e.g., IL‐2, IL‐6), thereby elevating overall immune competence (Lewis et al., 2019). In broilers, Khatun et al. (2020) reported that increasing dietary vitamin E reduced the expression of IFN and TNF‐α while upregulating TGF‐β1, a cytokine essential for T‐lymphocyte development and regulation of inflammatory responses.

#### Effects on fertility and reproduction

Vitamin E provides robust antioxidant protection to reproductive tissues, including egg yolks, spermatozoa, and developing embryos. In laying hens, dietary vitamin E supplementation is positively correlated with α‐tocopherol levels in eggs, thereby enhancing the oxidative stability of both hatching eggs and developing embryos. Elevated oxidative stress in the yolk sac membrane can impair hatchability, whereas dietary or *in ovo* vitamin E supplementation improves embryonic redox balance, enhances chick quality, and supports early post‐hatch growth, ultimately contributing to superior broiler performance (Meydani et al., 1988; Surai et al., 1999; Araújo et al., 2019). In males, combined supplementation with selenium, vitamin E, and vitamin C markedly improves semen antioxidant capacity, reduces lipid peroxidation, and stabilizes sperm quality. In a 90‐d trial, boars fed a diet containing 0.5 mg/kg selenium, 350 mg/kg vitamin C, and 70 mg/kg vitamin E exhibited an 85% increase in seminal antioxidant capacity and a 36% reduction in lipid peroxidation, resulting in significantly improved reproductive performance (Horky et al., 2016).

#### Effects on meat quality

Vitamin E plays a pivotal role in maintaining and improving meat quality by preventing oxidative degradation of muscle lipids and pigments. As a lipid‐phase antioxidant embedded in cellular and subcellular membranes, it enhances the oxidative stability of muscle tissue and deposited fats. Postmortem, PUFAs readily undergo oxidation, producing aldehydes, ketones, alcohols, esters, and acids that contribute to off‐flavors, cholesterol oxidation products, and meat discoloration due to the conversion of oxymyoglobin to metmyoglobin (Hao et al., 2025). By quenching lipid‐derived radicals, vitamin E delays both color and flavor deterioration, with particularly strong preservation effects observed in beef (Schaefer et al., 1995).

Continuous dietary supplementation with vitamin E increases α‐tocopherol enhancement in muscle tissues. When muscle α‐tocopherol concentrations reach approximately 3 μg/g, effective oxidative protection is achieved, delaying meat discoloration by inhibiting metmyoglobin formation and reducing lipid peroxidation, as reflected by lower thiobarbituric acid reactive substances (TBARS) values (Arnold et al., 1993). Notably, grass‐fed cattle tend to exhibit higher muscle α‐tocopherol levels than those finished on high‐energy grain diets (Silva et al., 2022).

In poultry, dietary supplementation of 200 mg/kg vitamin E has been shown to reduce TBARS formation by more than 80%, lower cholesterol‐oxidation products by 50%, and decrease secondary oxidation products such as aldehydes and ketones by approximately 50% (Barroeta, 2007). A meta‐analysis of 51 studies confirmed that vitamin E supplementation significantly increases muscle vitamin E content (*P* = 0.001) and reduces lipid peroxidation (*P* = 0.01) without altering PUFA composition, demonstrating its consistent improvement of chicken meat quality (Pompeu et al., 2018). In pork production, multiple studies report that dietary vitamin E at 100–200 mg/kg enhances oxidative stability and storage quality (Pettigrew and Esnaola, 2001). A meta‐analysis by Trefan et al. (2011) quantified the relationship between vitamin E supplementation and muscle α‐tocopherol accumulation, showing a nonlinear response with a maximum level of 6.4 μg/g. A minimum of 100 IU of dietary vitamin E is required to significantly suppress lipid oxidation, with each 1 μg/g increase in muscle α‐tocopherol lowering TBARS values by approximately 0.05. Subsequent studies further demonstrated that α‐tocopherol content correlates positively with meat redness (CIE a*), increasing by an average of 0.11 per mg of α‐tocopherol across storage periods, highlighting vitamin E's role in maintaining color stability (Boler et al., 2009; Trefan et al., 2010).

## BIOSYNTHESIS PATHWAY OF VITAMIN E AND ITS REGULATION

Vitamin E, collectively known as tocochromanols, occurs mainly in photosynthetic organisms. Each molecule consists of a polar chromanol headgroup linked to an isoprenoid side chain. Based on the degree of saturation in the side chain, tocochromanols are classified into tocopherols, tocotrienols, PC‐8, and tocomonoenols. Tocopherols possess a fully saturated phytyl side chain, whereas tocotrienols contain three additional *trans* double bonds. PC‐8 has a longer, unsaturated side chain similar to tocotrienols, while tocomonoenols carry only a single double bond (Tian et al., 2007; Sadre et al., 2010; Stacey et al., 2016). Differences in the number and position of methyl groups on the chromanol ring generate four isoforms, including α, β, γ, and δ, with PC‐8 existing naturally only in the δ‐form (Kamal‐Eldin and Appelqvist, 1996).

Tocopherols are ubiquitously synthesized across plant species and are particularly abundant in photosynthetic tissues and seeds. Tocotrienols, by contrast, have a narrower distribution, mainly in the endosperm of monocotyledonous species such as maize, barley, wheat, oats, rye, rice, oil palm, and coconut (Horvath et al., 2006; Falk and Munné‐Bosch, 2010). Although their bioavailability is generally lower than that of tocopherols (Hosomi et al., 1997), tocotrienols often exhibit superior antioxidant capacity, sometimes surpassing tocopherols in specific systems (Babura et al., 2017). This enhanced activity is likely due to the three additional double bonds that enhance their ability to scavenge lipid peroxyl radicals (Suzuki et al., 1993). Beyond antioxidation, tocotrienols display diverse physiological activities, including cholesterol‐lowering effects (Raederstorff et al., 2002), cardioprotection, and induction of cancer‐cell apoptosis (Sylvester and Shah, 2005).

PC‐8 was first identified in the leaves of the rubber tree and later found enriched in crops such as oilseed rape, tomato fruits, and the tubers of *Psidium guajava* var. alata (Goffman and Möllers, 2000; Zbierzak et al., 2009). The discovery of tocomonoenols arose from studies of organisms inhabiting extreme environments. In 1999, a novel vitamin E homolog was isolated from the eggs of Pacific salmon (*Oncorhynchus keta*) and designated marine‐derived tocopherol (MDT). This compound closely resembles α‐tocopherol but contains a single unsaturation near the terminal methyl group, marking the first discovery of such a molecule in an animal source (Yamamoto et al., 1999). Subsequent investigations revealed plant counterparts: In 1995, Japanese researchers detected a new vitamin E homolog with one double bond at the C11′ position in palm oil, naming it α‐tocomonoenol (α‐T1) (Matsumoto et al., 1995). α‐T1 has since been detected in palm, pumpkin, and sunflower seed oils (Matsumoto et al., 1995; Butinar et al., 2011; Hammann et al., 2015), as well as in aquatic photosynthetic organisms such as specific microalgae, where it can accumulate to high levels (Yamamoto et al., 2001; Dunlap et al., 2002; Gotoh et al., 2011).

Functional studies on tocomonoenols remain limited, but available evidence suggests that 12′‐α‐T1 may contribute to cold‐water adaptation in marine organisms (Yamamoto et al., 2001). Both 11′‐α‐T1 and 12′‐α‐T1 are bioavailable in humans (Yamamoto et al., 2001) and rodents (Gotoh et al., 2009; Beppu et al., 2018; Irías‐Mata et al., 2020). The precise biological roles of PC‐8 and tocomonoenols, however, remain largely unresolved and represent promising frontiers for future research.

### Biosynthesis pathway of vitamin E

**Figure 4:**
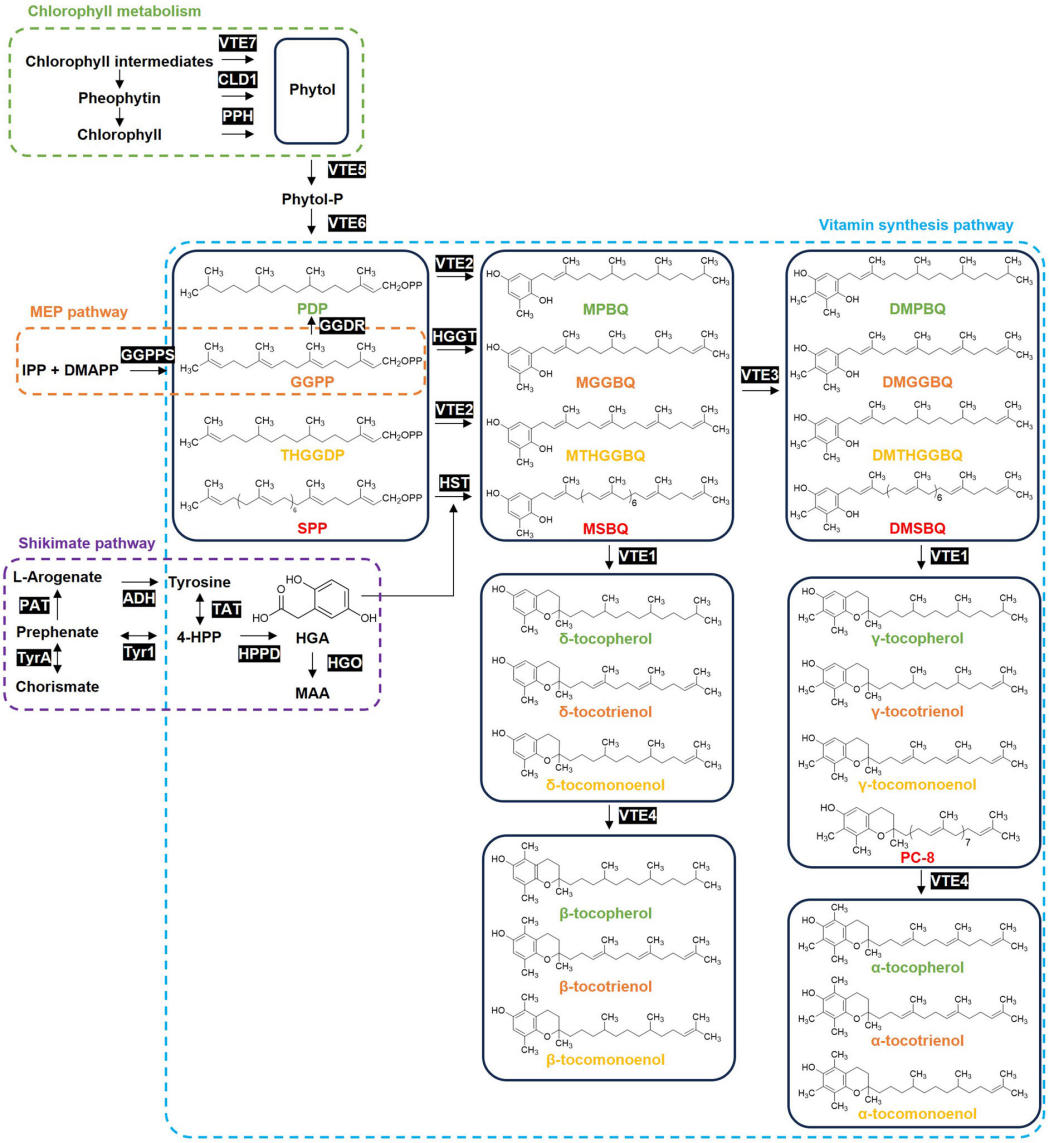
Integrated biosynthetic network and precursor origins of plant tocochromanol The diagram integrates the shikimate, methylerythritol phosphate (MEP), and chlorophyll‐degradation pathways that collectively supply precursors for tocochromanol biosynthesis. Homogentisate (HGA) is derived from tyrosine via the shikimate pathway, while phytyl diphosphate (PDP) and geranylgeranyl pyrophosphate (GGPP) originate from the MEP pathway or phytol recycling through chlorophyll metabolism. Key enzymatic steps (VTE1–VTE7, HPPD, HGGT, GGDR) and their associated intermediates are shown. Color‐coding differentiates metabolic modules, intermediates, and tocopherol derivatives, highlighting the compartmental and biochemical integration that underpins tocochromanol biosynthesis. Abbreviations of metabolites: DMAPP, dimethylallyl pyrophosphate; DMGGBQ, 2,3‐dimethyl‐5‐geranylgeranylbenzoquinol; DMPBQ, 2,3‐dimethyl‐6‐phytyl‐1,4‐benzoquinone; DMSBQ, 2,6‐dimethoxy‐1,4‐benzoquinone. 4‐HPP, 4‐hydroxyphenylpyruvate; DMTHGGBQ, 2,3‐dimethyl‐6‐tetrahydrogeranylgeranyl‐1,4‐benzoquinol; GGPP, geranylgeranyl pyrophosphate; HGA, homogentisate; IPP, isopentenyl diphosphate; MAA, 4‐maleyl‐acetoacetate; MGGBQ, 2‐methyl‐6‐geranylgeranyl‐1,4‐benzoquinol; MPBQ, 2‐methyl‐6‐phytyl‐1,4‐benzoquinol; MSBQ, 2‐methyl‐6‐solanesyl‐1,4‐benzoquinol; MTHGGBQ, 2‐methyl‐6‐tetrahydrogeranylgeranyl‐1,4‐benzoquinol; PC‐8, plastochromanol‐8. PDP, phytyl diphosphate; SPP, solanesyl diphosphate; THGGPP, tetrahydrogeranylgeranyl pyrophosphate. Abbreviations of enzymes: ADH, arogenate dehydrogenase; CLD1, chlorophyll dephytase 1; GGDR, geranylgeranyl diphosphate reductase; GGPPS, geranylgeranyl pyrophosphate synthase; HGGT, homogentisate geranylgeranyltransferase; HGO, homogentisate 1,2‐dioxygenase; HPPD, 4‐hydroxyphenylpyruvate dioxygenase; HST, homogentisate solanesyltransferase; PAT, prephenate aminotransferase; PPH, pheophorbide hydrolase; TAT, tyrosine aminotransferase; Tyr1, prephenate dehydrogenase; TyrA, chorismate mutase; VTE1, tocopherol cyclase; VTE2, homogentisate phytyltransferase; VTE3, 2‐methyl‐6‐phytyl‐1,4‐benzoquinol; VTE4, γ‐tocopherol methyltransferase; VTE5, phytol kinase; VTE6, phytyl phosphate kinase; VTE7, seed‐specific alpha/beta hydrolase.

The biosynthetic pathway of vitamin E is highly conserved among plants and algae and relies on two key precursors: Homogentisate (HGA), which donates the polar chromanol ring, and PDP or GGPP, which contributes the hydrophobic isoprenoid side chain. These two substrates condense within plastids to initiate the formation of tocopherols and tocotrienols (Figure 4).

#### Biosynthesis of precursor

HGA originates from the tyrosine degradation pathway, where tyrosine is first transaminated by tyrosine aminotransferase (TAT) to form 4‐hydroxyphenylpyruvate (HPP), which is then oxidized by 4‐hydroxyphenylpyruvate dioxygenase (HPPD) to yield HGA. In Arabidopsis, both AtTAT1 and HPPD are predominantly localized in the cytosol, indicating that HGA synthesis occurs partly or entirely outside the plastid, while the downstream synthesis of tocochromanols takes place within plastids. This spatial separation implies the existence of an as‐yet‐unidentified transporter that shuttles cytosolic HGA into plastids for tocopherol biosynthesis. Interestingly, the subcellular localization of HPPD varies among plant species (Pellaud and Mène‐Saffrané, 2017). In Arabidopsis (Wang et al., 2016) and carrot (Daucus carota; Garcia et al., 1997), HPPD resides in the cytosol, whereas in tomato, cotton, spinach, maize, and duckweed, it is plastid‐localized (Löffelhardt and Kindl, 1979; Fiedler et al., 1982; Siehl et al., 2014; Kim et al., 2021). Such variability suggests species‐specific compartmentation of the early biosynthetic steps, which may influence the efficiency of HGA supply and its integration into the vitamin E pathway.

The biosynthetic routes for tocopherol and tocotrienol side chains are both interconnected and distinct. PDP serves as the direct precursor for the saturated side chain of tocopherols. PDP can be synthesized *de novo* through the plastidial MEP pathway or derived from the phytol‐recycling pathway during chlorophyll degradation. In the latter route, free phytol is sequentially phosphorylated by phytol kinase (VTE5) and VTE6 to generate PDP in two steps (Valentin et al., 2006; Vom Dorp et al., 2015; Gutbrod et al., 2019). For tocotrienols, the corresponding prenyl donor is GGPP, which is likewise synthesized via the MEP pathway. Importantly, GGPP reductase (GGDR) can reduce GGPP to PDP, thereby linking the biosynthetic routes of tocopherols and tocotrienols (Keller et al., 1998; Tanaka et al., 1999). This metabolic interconnection ensures coordinated regulation between the formation of saturated and unsaturated side chains, allowing plants to flexibly adjust vitamin E composition in response to developmental or environmental cues.

Before 2006, the PDP used for tocopherol biosynthesis was thought to arise mainly from the direct reduction of GGPP by GGDR (Keller et al., 1998). However, accumulating evidence has revealed that chlorophyll metabolism is intimately coupled with vitamin E synthesis and that most of the phytyl moieties incorporated into tocopherols originate from chlorophyll degradation rather than de novo synthesis (Valentin et al., 2006; Gutbrod et al., 2019). Identifying the hydrolase responsible for releasing phytol or its derivatives from chlorophyll has therefore been key to resolving this biosynthetic connection.

Earlier studies characterized several enzymes associated with chlorophyll catabolism, including chlorophyllase (CLH) (Jacob‐Wilk et al., 1999; Tsuchiya et al., 1999), pheophytin pheophorbide hydrolase (PPH) (Schelbert et al., 2009), and chlorophyll dephytase 1 (CLD1) in Arabidopsis (Lin et al., 2016). Although CLH, PPH, and CLD1 can liberate phytol from chlorophyll or its demetallated forms, none of the single or multiple mutants in these genes produced a marked change in tocopherol content, and their overexpression only modestly elevated tocopherol levels. These findings suggested that the key α/β‐hydrolase driving phytol recycling for tocopherol biosynthesis had yet to be identified (Zhang et al., 2014).

A breakthrough came in 2022, when a genome‐wide association study (GWAS) identified VTE7, an enzyme localized to the chloroplast envelope, the primary site of tocopherol biosynthesis (Albert et al., 2022). Functional analyses showed that *VTE7* knockout reduced total seed tocopherol levels by approximately 55%, whereas *VTE7* overexpression in leaves increased tocopherol content 3.6‐fold, second only to the effect observed with *VTE2* overexpression (Collakova and DellaPenna, 2003). Co‐overexpression of *VTE7* and *VTE2* exhibited additive effects, confirming their complementary roles in phytyl incorporation. The discovery of *VTE7* thus revealed a direct and efficient phytol‐recycling route, establishing a molecular bridge between chlorophyll degradation and vitamin E formation. We propose that higher plants possess a coordinated “chlorophyll–catabolism–vitamin E‐biosynthesis” module. During leaf senescence and seed development, chlorophyll‐degradation signals, possibly mediated by brassinosteroids (BRs) (Chen et al., 2024), may simultaneously induce *VTE7* and core biosynthetic genes such as *VTE2*, redirecting carbon flux from photosynthetic pigments toward antioxidant production to mitigate oxidative stress. Testing this hypothesis will require time‐resolved tracking of chlorophyll intermediates and tocopherol levels to capture their coordinated dynamics.

#### Core synthesis pathway

The core steps of vitamin E biosynthesis take place within plastids and are catalyzed by a coordinated series of enzymes. The pathway begins with a condensation reaction catalyzed by HPT/VTE2, which couples HGA with PDP to form 2‐methyl‐6‐phytyl‐1,4‐benzoquinol (MPBQ), the key first intermediate in tocopherol formation. The *HPT* gene is conserved in all green plants and in several algal species, including the cyanobacterium *Synechocystis* (Collakova and DellaPenna, 2001; Savidge et al., 2002). In addition to using PDP, HPT can utilize tetrahydrogeranylgeranyl pyrophosphate (THGGPP) as a prenyl donor to produce 2‐methyl‐6‐tetrahydrogeranylgeranyl‐1,4‐benzoquinol (MTHGGBQ), a precursor of tocomonoenols in Arabidopsis seeds (Pellaud et al., 2018).

In monocotyledonous plants, HGGT is typically expressed specifically in seeds, catalyzing the condensation of HGA with GGPP to form 2‐methyl‐6‐geranylgeranyl‐1,4‐benzoquinol (MGGBQ), the precursor of tocotrienol biosynthesis (Cahoon et al., 2003; Yang et al., 2011). Because HGGT is structurally similar to VTE2, it can also utilize PDP to synthesize MPBQ, albeit with lower catalytic efficiency. Remarkably, barley HGGT can restore both tocopherol and tocotrienol production in Arabidopsis *vte2* mutants (Yang et al., 2011), underscoring the functional overlap between these enzymes. Another prenyltransferase, homogentisate solanesyltransferase (HST), condenses HGA with solanesyl diphosphate (SPP) to produce 2‐methyl‐6‐solanesyl‐1,4‐benzoquinol (MSBQ), the immediate precursor of PC‐8 (Sadre et al., 2006; Tian et al., 2007). The subsequent methylation and cyclization reactions transform these prenylated benzoquinones into the final tocochromanol products. MPBQ methyltransferase (MPBQ‐MT/VTE3) methylates MPBQ, MGGBQ, MSBQ, and MTHGGBQ to yield the dimethylated intermediates DMPBQ, DMGGBQ, DMSBQ (PQ‐9), and DMTHGGBQ (Cheng et al., 2003; Van Eenennaam et al., 2003). Both methylated and unmethylated intermediates can then undergo cyclization catalyzed by tocopherol cyclase (VTE1) to generate the δ‐ and γ‐forms of tocochromanols (Cheng et al., 2003). Finally, γ‐tocopherol methyltransferase (γ‐TMT/VTE4) methylates γ‐ and δ‐tocochromanols to produce α‐ and β‐tocochromanols, respectively (Shintani and Dellapenna, 1998; Bergmüller et al., 2003). Together, these reactions define the core biosynthetic network that diversifies tocochromanol structures, linking precursor availability and enzyme specificity to the compositional plasticity of vitamin E across plant species.

### Regulation of vitamin E biosynthetic pathways

#### Transcriptional regulation

Current understanding of transcriptional regulation in vitamin E biosynthesis remains limited, and most findings are still in the early stages of validation. Using weighted gene co‐expression network analysis (WGCNA), Du et al. (2024) identified EmWRKY13 as a potential negative regulator of the pathway. Overexpression of *EmWRKY13* in tobacco leaves reduced total vitamin E content by 23.37%, suggesting a repressive role in tocochromanol synthesis. Through mutant screening in Arabidopsis, Pellaud et al. (2018) found that the transcription factor WRINKLED 1 (WRI1) influences the accumulation of all tocopherol isoforms in seeds. A homolog of WRI1 in *Torreya grandis* also showed putative involvement in tocopherol regulation (Lou et al., 2019). However, these studies lack direct molecular evidence, such as target‐promoter binding assays or *in vivo* transcriptional validation, leaving the mechanistic roles of these factors unresolved.

A more definitive link was recently established in soybean. Through integrated metabolome‐genome association analysis (mGWAS) and molecular assays, Chu et al. (2023) demonstrated that the fatty‐acid‐responsive transcription factor GmZF351 directly activates several key genes in the vitamin E biosynthetic pathway, including *Gmγ‐TMT3*, *GmHPT*, *GmMPBQ‐MT1*, and *GmHPPD1*. GmZF351 specifically binds to E1E2 motifs, which are repetitive CT(G/C)(T/A)AA sequences within these promoters, as confirmed by yeast one‐hybrid (Y1H), electrophoretic mobility‐shift assay (EMSA), and dual‐luciferase reporter assays. Stable overexpression of *GmZF351* in transgenic soybean seeds markedly increased total and α‐tocopherol levels while upregulating the expression of multiple vitamin E biosynthetic genes. These findings reveal a transcriptional coordination module linking fatty‐acid metabolism and vitamin E biosynthesis, providing a mechanistic basis for the co‐improvement of oil quality and nutritional value. Nevertheless, several limitations remain. The upstream regulation of *GmZF351* remains unresolved. It is still unclear how environmental cues such as light, phytohormones, or oxidative stress modulate its transcription, stability, or activity. In addition, most functional evidence has been obtained from overexpression lines, without knockout validation using approaches such as CRISPR‐Cas9 to determine whether loss of *GmZF351* suppresses tocopherol synthesis under physiological conditions. In the absence of such genetic evidence, its *in vivo* necessity remains unverified.

A promising new direction lies in computationally guided promoter engineering. A recent study introduced a deep‐learning framework, Basenji2, capable of predicting gene‐expression levels from DNA sequences and proposing an “editing plasticity” metric that estimates the maximum expression change achievable through promoter editing (Qiu et al., 2025). Applying this approach, researchers identified regulatory elements across the maize genome and successfully used CRISPR‐Cas9 to edit the promoter of *ZmVTE4*, thereby enhancing its transcription and significantly increasing α‐tocopherol accumulation. This strategy partially circumvents the complexity of endogenous transcription factor networks and provides a robust alternative for rational optimization of gene expression.

Overall, transcriptional regulation of vitamin E biosynthesis remains an emerging field. Most proposed regulators have been inferred from omics correlations rather than direct molecular validation. Future work should integrate CRISPR‐Cas9 gene editing, chromatin immunoprecipitation sequencing (ChIP‐seq), yeast one‐hybrid, and *in vitro* binding assays to systematically confirm transcription‐factor interactions with the promoters of vitamin E biosynthetic genes. Equally important is to elucidate how these transcriptional modules integrate light signaling, hormonal cues (such as jasmonates), and oxidative stress into a coherent regulatory framework. Expanding research on negative regulators, environment‐responsive crosstalk, and tissue‐specific expression networks will ultimately enable the construction of a comprehensive transcriptional atlas for vitamin E biosynthesis in plants.

#### Environmental regulation

As a key lipid‐soluble antioxidant, vitamin E accumulates to high levels during both biotic and abiotic stress, reflecting its essential role in plant environmental adaptation (Bao et al., 2020). Conversely, plants deficient in vitamin E are markedly more sensitive to stress treatments (Havaux et al., 2005; Maeda et al., 2006; Abbasi et al., 2007; Muñoz and Munné‐Bosch, 2019), underscoring its central function in maintaining redox homeostasis and stress resilience.

Elevated temperature accelerates chlorophyll turnover (Lin et al., 2016), generating GGPP, which can be reduced to PDP, a key substrate for tocopherol biosynthesis (Mène‐Saffrané and DellaPenna, 2010). Using high‐throughput extraction and high‐performance liquid chromatography (HPLC) analysis, Bao et al. (2020) systematically examined how heat stress (37°C) regulates vitamin E metabolism in Arabidopsis leaves. Tocopherol accumulation increased dramatically and displayed strong temporal and isomer‐specific patterns. Similarly, vitamin E‐deficient maize mutants exhibit impaired growth at low temperature (Maeda et al., 2006). In tomato, silencing of *VTE5* in the chlorophyll‐recycling pathway causes severe chlorosis and drastically reduced α‐tocopherol levels under combined heat and high‐light stress (Spicher et al., 2017). Collectively, these findings suggest that under high temperature, plants redirect chloroplastic carbon flux from pigment metabolism toward tocopherol biosynthesis to counteract oxidative damage.

Under high irradiance or combined heat‐light stress, tocopherols, particularly α‐tocopherol, are strongly induced to protect photosystems from singlet‐oxygen damage and to stabilize thylakoid membranes (Kruk et al., 2005). Arabidopsis mutants lacking α‐tocopherol are hypersensitive to elevated temperatures (Kobayashi and DellaPenna, 2008), confirming its indispensable antioxidant function. Combined high‐light and low‐temperature stress causes even more severe oxidative injury, characterized by intense lipid peroxidation, a phenotype especially pronounced in *vte1* mutants (Havaux et al., 2005). Moreover, in species such as alfalfa and lettuce, high light induces transcriptional upregulation of HPPD, a key enzyme in tocopherol biosynthesis (Ren et al., 2011; Jiang et al., 2017). This conserved response likely represents an adaptive compensation mechanism that enhances redox buffering capacity under photooxidative stress.

Several core genes in the vitamin E pathway, notably *HPPD*, play crucial roles in drought tolerance by mitigating oxidative damage (Ren et al., 2011; Jiang et al., 2017; Kim et al., 2021). In rice, *OsVTE1* expression increases markedly under water deficit (Ouyang et al., 2011). Functional studies provide direct evidence: Heterologous overexpression of *AtVTE1* in tobacco enhances drought tolerance by reducing lipid peroxidation, hydrogen peroxide accumulation, and electrolyte leakage (Liu et al., 2008). Similarly, overexpression of *MsVTE4* in alfalfa increases α‐tocopherol and total tocopherol levels, alleviating oxidative injury and improving drought resilience (Ma et al., 2020). These results collectively demonstrate that boosting vitamin E biosynthetic capacity is an effective strategy for improving drought tolerance.

In salinity responses, tocopherols mitigate oxidative damage and help maintain ionic homeostasis. Silencing *VTE2* in tobacco sharply reduces total tocopherol levels and disrupts ion balance, while *VTE4*‐silenced plants display reduced stress resistance (Abbasi et al., 2007). Conversely, overexpression of *AtVTE4* significantly decreases superoxide accumulation, lipid peroxidation, and ion leakage under salt stress (Jin and Daniell, 2014). Comparative analyses using Arabidopsis mutants *vte1* (deficient in α‐ and γ‐tocopherol) and *vte4* (γ‐tocopherol overaccumulation) revealed that α‐tocopherol exerts stronger regulatory control over chloroplast biogenesis and reactive oxygen species (ROS) signaling than γ‐tocopherol, playing a dominant role in salt adaptation (Surówka et al., 2020). Based on these insights, genes such as *AtVTE2* and *AtVTE4* have been introduced into crops to enhance resistance not only to salinity but also to heavy‐metal stress (Upadhyaya et al., 2021).

Overall, vitamin E biosynthesis functions as a dynamic regulatory network finely tuned by environmental signals. At its core, the pathway is governed by transcriptional control mediated by incompletely characterized transcription factors and co‐adjusted with lipid metabolism. More importantly, environmental signals such as light, heat, drought, and salinity strongly modulate its activity, while vitamin E itself feeds back to stabilize redox balance and maintain membrane integrity. Future research should aim to construct a systems biology model that integrates environmental inputs, signal transduction, transcriptional regulation, metabolic fluxes, and physiological outcomes. Such a framework will illuminate how vitamin E functions as both a biochemical shield and a regulatory node at the heart of plant stress adaptation.

## VITAMIN E PRODUCTION TECHNOLOGIES

### Natural sources, extraction, and characteristics of vitamin E

Natural vitamin E is abundant in vegetable oils, which constitute its major natural source (Pandya et al., 2019). Industrial production therefore relies largely on the deodorizer distillates generated during vegetable oil refining and on processing by‐products from oilseed crops. Several extraction techniques have been developed, including saponification‐extraction (Knecht et al., 2015), solvent extraction (Buczenko et al., 2003), supercritical‐fluid extraction (de Lucas et al., 2002; Luan et al., 2006), molecular distillation (Luan et al., 2006), and ion‐exchange adsorption (Wu et al., 2016).

The vitamin E content of deodorizer distillates varies widely among oil sources. Soybean‐oil distillate, containing about 2%–15% natural vitamin E, is currently the principal industrial feedstock (Beal et al., 1956). Before extraction, distillates are pretreated to remove fatty‐acid impurities, typically through esterification or saponification. In esterification, free fatty acids are converted to methyl esters using short‐chain alcohols such as methanol; subsequent cooling crystallization removes sterols, allowing separation of methyl esters from vitamin E (Muñoz and Munné‐Bosch, 2019). In saponification, alkali treatment converts free fatty acids to soap (sodium salts), after which the vitamin E‐rich unsaponifiable fraction is recovered by solvent extraction and purified (Knecht et al., 2015). These processes yield concentrated natural vitamin E products that retain high bioactivity. Compared with synthetic forms, natural vitamin E exhibits superior absorption and physiological efficacy.

However, several challenges constrain its large‐scale production. The cellular concentration of vitamin E in plants is extremely low (microgram per gram range) (DellaPenna, 2005), resulting in limited yield per unit biomass. Tocochromanol composition varies greatly among species (Li et al., 2011), and agricultural production is restricted by cultivation cost, climate dependence, and the complexity of downstream purification. Moreover, large‐scale monoculture for oil production may pose risks to biodiversity. These factors collectively limit the ability of plant‐based sources to meet global market demand sustainably.

Notably, the proportion of the most biologically active stereoisomer, (R,R,R)‐α‐tocopherol, is relatively low in natural plant oils, which instead contain higher amounts of the less active γ‐ and δ‐tocopherols (Müller et al., 2022). The binding affinity of the human α‐TTP for different forms follows the order of (R,R,R)‐α‐tocopherol (100%) > R,R,R‐β (38%) > R,R,R‐γ (9%) > R,R,R‐δ (2%) (Hosomi et al., 1997). To enhance biological activity, natural tocopherols are often methylated to generate α‐tocopherol (Katsui, 1965; Baldwin et al., 1990; Lechtken et al., 1990). The acetate ester, D‐α‐tocopheryl acetate, is now the predominant form used in dietary supplements and fortified foods (Müller et al., 2022). Produced by esterification of the highly active (R,R,R)‐α‐tocopherol, this derivative offers enhanced oxidative stability during storage and processing. Although free tocopherols possess stronger intrinsic antioxidant activity, esters such as acetates are efficiently hydrolyzed by lipases *in vivo* to release active tocopherol, ensuring full biological efficacy. Owing to their excellent stability and bioavailability, tocopherol esters such as acetates and succinates are widely used in food additives, pharmaceuticals, cosmetics, animal feed, and nutritional supplements. With the growing global demand for vitamin E, the continued development of high‐value downstream applications and expanded utilization remain a central challenge for the industry.

### Chemical synthetic process and stereochemical optimization

**Figure 5:**
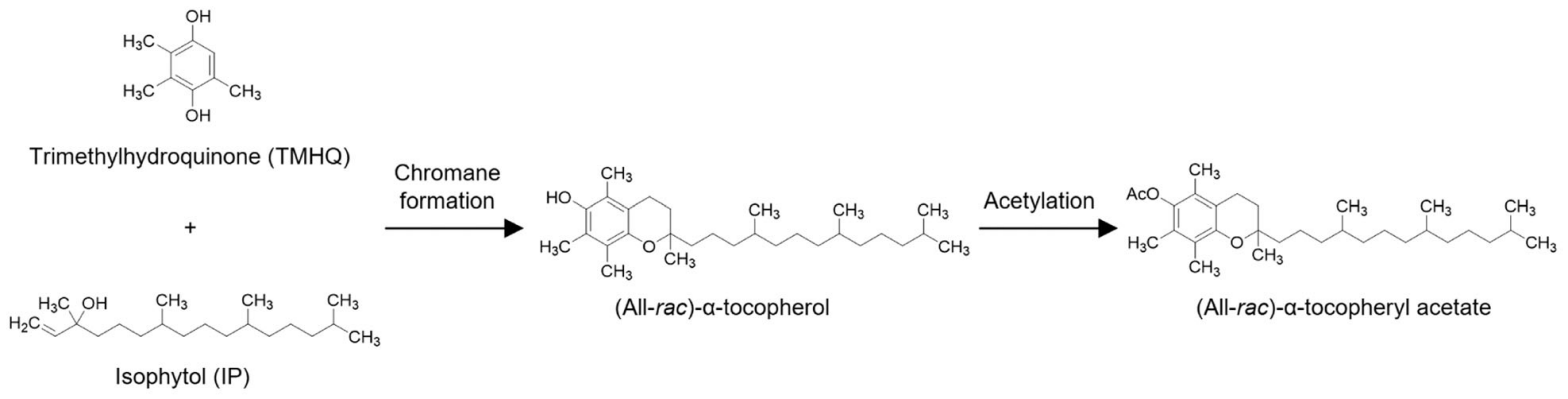
Final reaction steps in the industrial synthesis of (all‐rac)‐α‐tocopheryl acetate The process involves Friedel‐Crafts condensation of trimethylhydroquinone (TMHQ) and isophytol (IP) to form a chromane ring, followed by cyclization to yield all‐rac‐α‐tocopherol. The final acetylation step produces α‐tocopheryl acetate, a more stable ester form widely used in commercial vitamin E formulations.

With the rising global demand for vitamin E, large‐scale chemical synthesis has become the dominant production route due to its structural controllability, high yield, and cost efficiency. Today, more than 80% of the world's vitamin E supply is produced synthetically, primarily for use in animal feed formulations, where consistent quality and low production cost are critical. The history of synthetic vitamin E dates back to 1938, when the first successful condensation of trimethylhydroquinone (TMHQ) with phytyl bromide yielded all‐rac‐α‐tocopherol, the fully racemic form of α‐tocopherol (Karrer et al., 1938). Over subsequent decades, extensive optimization of the reaction sequence improved overall yields to over 95% (Netscher, 2007). In modern industrial practice, the synthesis of all‐rac‐α‐tocopherol and its major derivative, α‐tocopheryl acetate, relies on two key intermediates, TMHQ and isophytol (IP) (Bonrath et al., 2007; Figure 5). These precursors are readily obtainable from petrochemical feedstocks, allowing continuous production with high reproducibility and scalability.

The process involves the condensation of TMHQ with IP via a Friedel‐Crafts alkylation and cyclization sequence to yield all‐rac‐α‐tocopherol, followed by acetylation to produce the more stable α‐tocopheryl acetate. The core step of industrial vitamin E synthesis is the Friedel‐Crafts condensation reaction, which proceeds through several sequential stages. The first stage is Friedel‐Crafts alkylation, where TMHQ reacts with IP in the presence of Lewis and/or Brønsted acid catalysts, producing an alkylated intermediate. This intermediate then undergoes a cyclization reaction, forming free all‐rac‐α‐tocopherol, which can subsequently be esterified, for example, to generate all‐rac‐α‐tocopheryl acetate. The acetylation step is typically catalyzed by acids or bases under either continuous or batch operation, using acetic anhydride as the acylating agent, with the by‐product acetic acid removed by distillation (Müller et al., 2022).

In nature, α‐tocopherol exists exclusively in the single (R,R,R) stereoisomeric form. In contrast, the condensation of TMHQ with racemic IP yields all‐rac‐α‐tocopherol, an equimolar mixture of eight stereoisomers (RRR, RSR, RRS, RSS, SRR, SSR, SRS, and SSS). These isomers differ significantly in their absorption, transport, and metabolic utilization *in vivo*, a disparity governed primarily by the α‐TTP. This 32 kDa cytosolic protein, first described by Catignani in 1975 (Catignani, 1975; Sato et al., 1991), exhibits a high degree of stereoselectivity, preferentially binding and efficiently transporting (R,R,R)‐α‐tocopherol with 100% binding efficiency. Other naturally occurring 2R‐configured isomers, such as RSR and RRS, can be transported but with substantially lower efficiency, whereas synthetic 2S‐configured isomers, including SRR, SSR, SRS, and SSS, are poorly recognized by α‐TTP and thus have negligible bioavailability (Weiser et al., 1996). As a result, the natural (R,R,R) form remains the most biologically potent and nutritionally valuable configuration.

Given the superior activity and high market value of (R,R,R)‐α‐tocopherol, the development of stereoselective synthetic routes has become a central research focus. One promising strategy involves employing enantioenriched isoprenoid side chains, often derived from naturally occurring (E,R,R)‐phytol (Eggersdorfer et al., 2012). Contemporary research into (R,R,R)‐α‐tocopherol synthesis has centered on several complementary approaches. One line of development utilizes biocatalytic or enzyme‐mediated reactions, exploiting the inherent stereoselectivity of enzymes to construct the required chiral centers (Stocker et al., 1993). Another relies on chiral‐pool or auxiliary‐based methods, assembling the molecule from optically pure precursors with pre‐defined stereochemistry (Ratsch et al., 2021). A third avenue focuses on asymmetric catalysis, in which chirality is introduced during key bond‐forming steps to achieve high stereochemical precision (Termath et al., 2014; Uyanik et al., 2014).

Recent progress in asymmetric catalysis has been particularly notable. In 2020, a novel iridium‐catalyzed ring‐opening reaction of cyclobutanols was developed, providing a direct route to construct the chiral center of vitamin E and solving a long‐standing challenge in its total synthesis (Ratsch et al., 2021). Further breakthroughs have been achieved through enantioselective hydrogenation for generating chiral isoprenoid side chains. Advanced iridium catalyst systems now enable asymmetric hydrogenation of non‐activated C=C bonds with exceptional enantioselectivity (ee > 98%) and near‐quantitative conversion (Takaya et al., 1987; Schmid et al., 1996; Wang et al., 2008; Woodmansee et al., 2010; Bonrath et al., 2021). Together, these methodological innovations have transformed the synthesis of (R,R,R)‐α‐tocopherol from a classical petrochemical process into a model of precision asymmetric catalysis. The iridium‐based method, in particular, has enabled the total synthesis of (2 R)‐α‐tocopherol, effectively bridging industrial scalability with the molecular fidelity of biomimetic chemistry.

### Biotechnological synthesis

**Figure 6:**
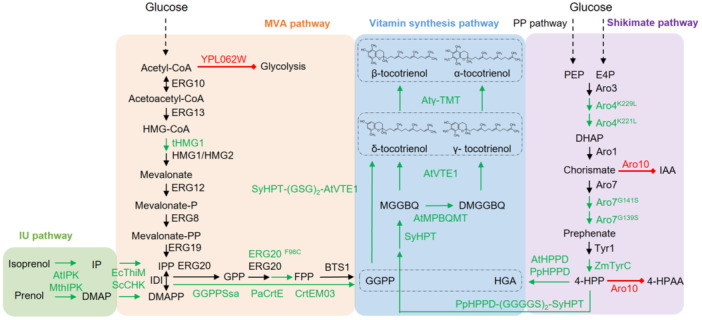
Biosynthetic pathway of tocotrienols in engineered microbial cell factories The MVA pathway module is marked in the orange box. The shikimate pathway module is marked in the purple box. The vitamin synthesis pathway module is marked in the blue box. Black arrows represent single‐step reactions. Double‐headed arrows represent reversible reactions. Dotted arrows represent multiple enzymatic steps. Enzymes shown in green represent the strategies used in existing research to enhance expression, while the enzymes highlighted with a red background represent knockout strategies employed in current research (Shen et al., 2020; Sun et al., 2020; Jiao et al., 2022, 2023; Xiang et al., 2025). Abbreviations of metabolites: 4‐HPAA, 4‐hydroxyphenylacetaldehyde; DHAP, 7P‐2‐dehydro‐3‐deoxy‐D‐arabino‐heptonate; DMAP, dimethylallyl phosphate; E4P, D‐erythrose‐4‐phosphate; FPP, farnesyl pyrophosphate; GPP, geranyl diphosphate; IAA, indole acetaldehyde; IP, isopentenyl phosphate; PEP, phosphoenolpyruvate. Abbreviations of enzymes: Aro1, pentafunctional AROM protein; Aro10, phenylpyruvate decarboxylase; ARO3, 3‐deoxy‐d‐arabino‐heptulosonate‐7‐phosphate (DAHP) synthase; Aro4, 3‐deoxy‐d‐arabino‐heptulosonate‐7‐phosphate (DAHP) synthase; Aro7, chorismate mutase; Aro8, aromatic aminotransferase I; Aro9, aromatic aminotransferase II; AtIPK, isopentenyl phosphate kinase from *Arabidopsis thaliana*; BTS1, farnesyltranstransferase. YPL062W, a dubious open reading frame whose deletion increased isoprenoid production; CrtE03M, the C81T variant of GGPP synthase from *Xanthophyllomyces dendrorhous*; EcThiM, hydroxyethylthiazole kinase from *E. coli*; ERG10, acetyl‐CoA C‐acetyltransferase; ERG12, mevalonate kinase; ERG13, 3‐hydroxy‐3‐methylglutaryl‐CoA (HMG‐CoA) synthase; ERG19, diphosphomevalonate decarboxylase; ERG20, farnesyl diphosphate synthetase; ERG8, phosphomevalonate kinase; ERG9, squalene synthetase; HPPD, 4‐hydroxyphenylpyruvate dioxygenase; IDI, isopentenyl diphosphate delta‐isomerase; MthIPK, isopentenyl phosphate kinase from *Methanothermobacter thermautotrophicus*; ScCHK, choline kinase from *Saccharomyces cerevisiae*; tHMG1, truncated HMG‐CoA reductase.

While chemical synthesis remains the dominant industrial route for vitamin E production, its inherent limitations, including the generation of stereoisomers with low biological activity, reliance on petrochemical feedstocks, and high energy and environmental costs, constrain its long‐term sustainability. Likewise, natural extraction suffers from low yield, high production cost, and dependence on agricultural and climatic factors. These constraints collectively define the current upper limit of global vitamin E supply. Recent advances in computational biology, systems biology, synthetic biology, and protein engineering have opened new possibilities for the de novo biosynthetic design of complex plant natural products, including vitamin E. Microbial cell factories, in particular, offer several advantages: Independence from climate conditions, scalability, environmentally friendly production, and a low carbon footprint (Figure 6).

The first milestone in microbial vitamin E biosynthesis was reported in 2008 by Christoph Albermann's group. For the first time, multiple key enzymes from different species were heterologously expressed in the non‐photosynthetic bacterium *Escherichia coli*, including HPPD, geranylgeranyl diphosphate synthase (CrtE), geranylgeranyl diphosphate reductase (Ggh), HPT, and tocopherol cyclase (Cyc). This engineered strain successfully constructed a complete biosynthetic pathway, enabling the *de novo* production of δ‐tocotrienol, though with a low yield of only 15 μg/g dry cell weight (DCW) (Albermann et al., 2008). This study confirmed the feasibility of synthesizing vitamin E compounds in non‐photosynthetic organisms and identified key limiting factors: the intermediate metabolite MGGBQ accumulated intracellularly in large quantities (approximately 15 times the final product), it was prone to oxidation and polymerization, and its potential location in the outer cell membrane potentially hindered its accessibility to the cytoplasmic cyclase enzyme, resulting in the low yield (Oram et al., 2001; Gruszecki and Strzałka, 2005).

Since plasmid‐carrying strains exhibited significant growth inhibition and severe plasmid loss (approximately 60% of cells lost the plasmid) during later culture stages, subsequent research adopted a chromosomal integration strategy to replace high‐copy‐number plasmids for improved stability and yield (Ghanegaonkar et al., 2013). The key genes of the synthesis pathway were integrated as independent units into specific loci of the *E. coli* genome, successfully creating plasmid‐free engineered strains. These strains not only achieved 100% genetic stability but also increased the MGGBQ production to 604 μg/g DCW, representing an approximately 86% improvement over the corresponding plasmid‐based system. The study further identified the supply of geranylgeranyl pyrophosphate (GGPP) as the primary rate‐limiting step. By strengthening the MEP pathway through metabolic engineering, including the integration of the isopentenyl pyrophosphate isomerase (*idi*) gene and replacing the native promoter of the *dxs* gene with a strong T5 promoter, the MGGBQ production was ultimately increased substantially to 1,425 μg/g DCW.

Given its superior tolerance to isoprenoid precursors, *Saccharomyces cerevisiae* was considered an ideal chassis for synthesis. Sun et al. (2020) first constructed a *de novo* biosynthetic pathway for δ‐tocotrienol in this yeast. A baseline engineered strain was created by introducing three heterologous reactions (HPPD, HPT, VTE1), but no product was detected due to insufficient endogenous GGPP supply. To address this, the research team strengthened the mevalonate (MVA) pathway (by overexpressing a truncated version of HMG‐CoA reductase, tHMG1) and introduced a heterologous GGPP synthase with high catalytic efficiency. This bypassed the farnesyl pyrophosphate (FPP) branch point, directly and efficiently utilizing the MVA pathway substrates isopentenyl pyrophosphate (IPP) and dimethylallyl pyrophosphate (DMAPP) for GGPP synthesis. The resulting engineered strain successfully produced 1.39 mg/L of δ‐tocotrienol. Subsequently, optimization of medium components using response surface methodology (RSM) increased the yield to 3.56 mg/L; further optimization of the process in fed‐batch fermentation ultimately achieved a yield of 4.10 mg/L.

The research by Yu Hongwei's team in *S. cerevisiae* was more systematic (Shen et al., 2020; Jiao et al., 2022, 2023). By introducing key enzymes from Arabidopsis and cyanobacteria, they successfully constructed a complete tocotrienol synthesis pathway enabling the production of a mixture of α‐, β/γ‐, and δ‐tocotrienols (Shen et al., 2020). Metabolic analyses indicated that the HPT derived from the cyanobacterium *Synechocystis* sp. PCC6803 played a critical role in pathway efficiency, likely by influencing the conversion of homogentisate to MGGBQ. Concurrently, the authors found that the N‐terminal chloroplast transit peptides of plant‐derived enzymes, including MPBQMT, TC, and γ‐TMT, hindered their function in yeast. Computational prediction and removal of these targeting peptides significantly enhanced enzyme activity; for example, MPBQMT activity increased approximately ninefold. Furthermore, the study systematically increased precursor supply by strengthening the shikimate and mevalonate pathways. Shikimate pathway flux was increased through the introduction of feedback inhibition‐insensitive mutants Aro4^K229L^ and Aro7^G141S^, expression of a heterologous tyrosine dehydrogenase (*TyrC*), and deletion of the aromatic amino acid degradation gene *ARO10*. In parallel, mevalonate pathway flux was strengthened by deleting *YPL062W* to enhance acetyl‐CoA availability and introducing a directed‐evolution‐derived GGPP synthase mutant CrtE^03M^ with high catalytic activity.

To alleviate growth inhibition caused by product accumulation, the team developed an innovative cold‐shock‐triggered temperature control system. This system was based on the temperature‐sensitive transcriptional activator Gal4M9. Upon activation at low temperature, it powerfully initiated the expression of all downstream biosynthetic genes via a positive feedback loop. This design required only a 5‐h cold‐shock treatment to efficiently trigger the production phase. Afterward, fermentation resumed at the normal 30°C, ultimately achieving a total tocotrienol production (including various isomers) of 320 mg/L in high‐density fermentation.

Building upon this work, subsequent studies further alleviated metabolic repression by deleting transcriptional repressors such as MOT3 and ROX1 and improved redox balance by overexpressing the mitochondrial NADH kinase *POS5*. To address intracellular product toxicity and enhance yield, a two‐phase extraction fermentation strategy was implemented, with olive oil identified as the optimal extraction phase (Jiao et al., 2022). Concurrently, active product secretion was promoted by screening and overexpressing endogenous ATP‐binding cassette (ABC) transporters, including *Pdr11p* and *Yol075cp*, which increased the secretion ratio of the tocotrienol mixture to 73.66%. As a result of these combined strategies, the final engineered strain achieved a total tocotrienol of 82.68 mg/L. In a subsequent study, the addition of the cyclodextrin derivative 2‐HP‐β‐CD as a trapping agent, together with transporter overexpression, further elevated δ‐tocotrienol production to 211.56 mg/L, accompanied by a remarkably high secretion ratio of 85.6% (Jiao et al., 2023).

The most recent study by Xiang et al. (2025) shifted the production host to *Yarrowia lipolytica*, a yeast naturally enriched in acetyl‐CoA and NADPH, whose intracellular lipid bodies can serve as storage compartments for hydrophobic products. By fusing the key enzymes SyHPT and AtVTE1 with an optimal linker peptide ((GSG)_2_) to promote a substrate channeling effect for the intermediate, the yield was increased from 102.8 mg/L to 137.9 mg/L. Subsequently, rational design of the rate‐limiting enzyme SyHPT yielded a K77Y mutant with 67% higher catalytic activity than the wild‐type. Integrating these metabolic engineering, enzyme engineering strategies, and multi‐copy genomic integration, the resulting optimal strain, cultivated in a 5‐L bioreactor under optimized controlled fed‐batch fermentation, ultimately achieved a δ‐tocotrienol production of 466.8 mg/L. Most recently, Xu et al. (2026) further advanced the *Y. lipolytica* platform by implementing a synthetic isopentenol utilization pathway (IUP) alongside the native mevalonate (MVA) pathway to bypass endogenous metabolic regulation and efficiently boost geranylgeranyl pyrophosphate (GGPP) supply. To overcome product secretion and stability bottlenecks, they identified and overexpressed four endogenous membrane transporters, such as the quinate permease YALI0D18876g, and implemented an antioxidant‐assisted fermentation strategy using butylated hydroxytoluene (BHT) to prevent oxidative degradation. Additionally, by developing a PpHPD‐SyHPT fusion to enhance enzyme solubility and truncating the N‐terminal signal peptide of AtTC, the engineered strain ultimately achieved a record δ‐tocotrienol titer of 617.23 mg/L in a 2L bioreactor, representing the highest level reported to date for microbial tocotrienol production.

### Bio‐hybrid methods

Another important innovation came from Tiangang Liu's group at Wuhan University, who developed a semichemical synthetic route to vitamin E that bridges microbial fermentation and chemical catalysis − the “bio‐hybrid approach” (Tian et al., 2020; Ye et al., 2022). This method abandons reliance on microbial *de novo* synthesis of the complete vitamin E molecule; instead, it strategically dissects the synthesis into two advantageous steps: First, efficient microbial production of a key intermediate via a cell factory, followed by its conversion to the final product through concise and efficient chemical catalysis. By integrating the selectivity of biological synthesis with the robustness and scalability of chemical methods, this bio‐hybrid approach offers a particularly effective solution for the production of structurally complex molecules such as vitamin E.

**Figure 7:**
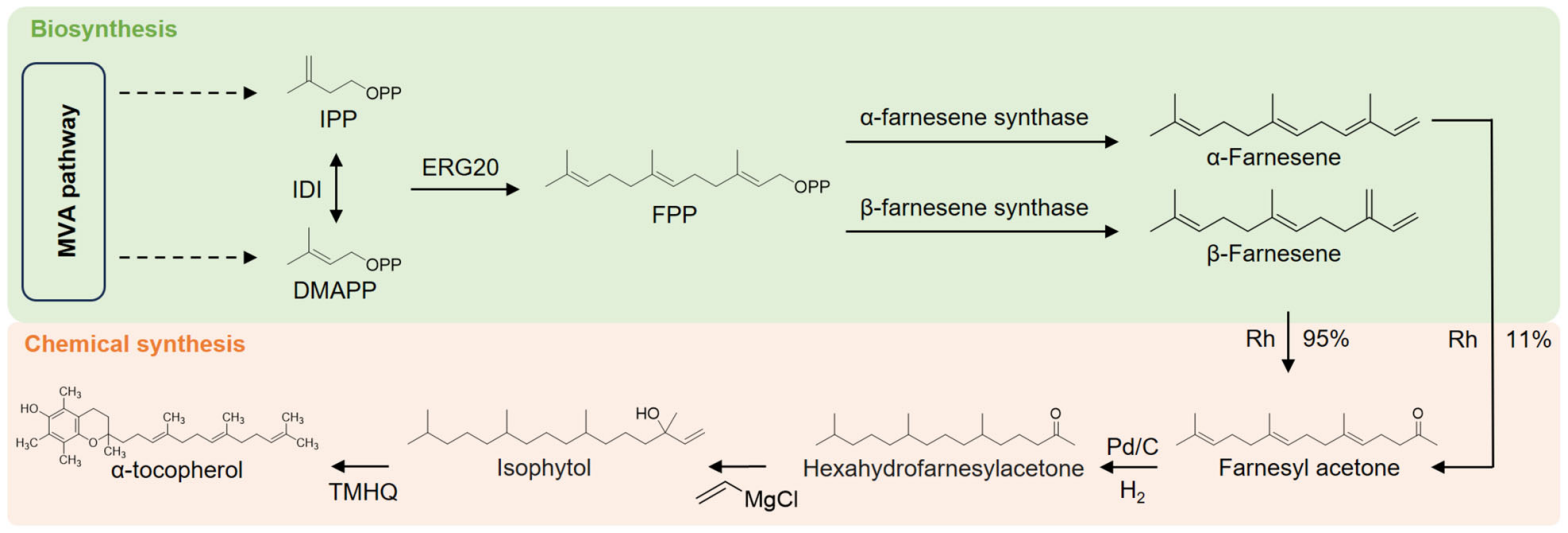
Synthetic pathway of the bio‐hybrid method The biosynthetic pathway is enclosed in a green box, while the chemical synthesis route is highlighted in an orange box.

The core of this hybrid route lies in using microbially fermented farnesene, a renewable isoprenoid that can be efficiently secreted by engineered microbes without compromising cell growth (Meadows et al., 2016), as a key intermediate for IP synthesis (Zhu et al., 2014). Farnesene is then efficiently converted to isophytol, the direct precursor of vitamin E, through a concise three‐step chemical process. Condensation of isophytol with trimethylhydroquinone then yields α‐tocopherol (Figure 7). This strategy dramatically simplifies the conventional seven‐step chemical synthesis of isophytol, which typically involves hazardous steps like high‐pressure alkyne reactions and demands specialized equipment. By contrast, the bio‐hybrid route reduces the synthesis to three mild and safer steps, achieving an overall yield of approximately 92%. As a result, this approach is economically competitive with the most efficient chemical synthesis routes while offering clear advantages in safety, sustainability, and process simplicity.

The process integrates biosynthesis (green panel) and chemical synthesis (orange panel). In the biological phase, the mevalonate (MVA) pathway generates isopentenyl pyrophosphate (IPP) and dimethylallyl pyrophosphate (DMAPP), which are converted to farnesyl pyrophosphate (FPP) by ERG20 (farnesyl diphosphate synthetase), and subsequently to α‐farnesene or β‐farnesene by respective synthases. In the chemical phase, farnesene is converted to farnesyl acetone via Rh‐catalyzed oxidation, followed by hydrogenation to hexahydrofarnesylacetone, Grignard reaction to isophytol, and final condensation with TMHQ to yield α‐tocopherol.

The success of this route is built on a solid research foundation. Pioneering work by Zhu et al. (2014) laid the groundwork for efficient farnesene production. They reconstructed the MVA pathway *in vitro*, avoiding interference from intracellular metabolic networks, precisely identifying isopentenyl diphosphate isomerase (Idi) as the key rate‐limiting enzyme, and detailed kinetic parameters were characterized. Rational metabolic engineering of *E. coli* based on optimal enzyme ratios enabled efficient conversion of glucose and glycerol into farnesene, reaching a titer of 1.1 g/L, approaching the theoretical maximum yield of 0.2 kg farnesene per kg glucose.

The stereochemistry of farnesene proved to be a decisive factor in conversion efficiency: α‐farnesene resulted in only 11% yield of farnesylacetone, whereas β‐farnesene achieved a conversion rate as high as 95%. Since *E. coli* predominantly produces α‐farnesene (approximately 95% purity as verified by NMR), the team turned to an engineered yeast strain, *S. cerevisiae* T16, which overexpressed tHMG1, FgJ03939, and ERG20 to enhance isoprenoid flux. They further identified β‐farnesene synthase from *Matricaria chamomilla* var. recutita as the most active catalyst, and subsequent directed‐evolution optimization of this enzyme produced a highly efficient β‐farnesene synthesis system. Finally, an extremely high farnesene production of 55.4 g/L was achieved in the fermenter, providing a cost‐effective raw material guarantee for industrial applications (Ye et al., 2022).

To further enhance economic viability, the team also developed a co‐production strategy for farnesene and the high‐value product lycopene. Although competition for the common precursor FPP leads to a slight decrease in farnesene yield, the co‐produced lycopene (market price ~$2,000/kg) is sufficient to cover the fermentation costs. This renders farnesene production nearly “cost‐free”, requiring only consideration of subsequent separation costs, thereby significantly boosting the overall process's market competitiveness.

It is important to note that while the upstream of this hybrid process revolutionarily employs synthetic biology, its downstream chemical synthesis steps, particularly those determining stereochemistry, still rely on classical non‐stereoselective methods. Consequently, the final product is the racemic all‐rac‐α‐tocopherol, not the single natural (R,R,R)‐α‐tocopherol stereoisomer. In essence, this represents a more advanced and sustainable chemical synthesis method, rather than a full biological synthesis aimed at producing a single stereoisomeric natural product.

Nonetheless, this integrated bio‐hybrid process, which combines synthetic‐biology‐based precursor biosynthesis with fine chemical catalysis, exhibits significant advantages in efficiency, safety, and environmental sustainability. By replacing hazardous intermediates and reducing energy consumption, it cuts CO_2_‐equivalent emissions by roughly 50%. The process was industrialized by Nenter and Co. in Jingzhou, Hubei Province, in the world's first plant using this technology, commissioned in 2017 with an annual capacity of 30,000 tons. The innovation stabilized global vitamin E supply and reduced its market price from approximately US$ 20 to US$ 10 per kg, setting a landmark example of how synthetic biology can transform and decarbonize traditional chemical manufacturing.

Tocotrienols have not yet achieved commercial‐scale production due to the complexity of their synthesis steps, coupled with low yield and purity. This current situation has driven research to focus on in‐depth exploration of their biotechnological synthesis, aiming to bridge the gap left by chemical synthesis methods. In recent years, significant progress has been made in constructing complete tocotrienol biosynthetic pathways in various microbial systems, such as *E. coli*, *S. cerevisiae*, and *Y. lipolytica*, with notable improvements in target product titer through strategies like metabolic engineering and directed evolution. On the other hand, all‐rac‐α‐tocopherol has reached mature industrial production and dominates the market. However, there remains a need for complementary biotechnological synthesis methods capable of producing enantiomerically pure natural forms to meet market demands. Key questions remain regarding how biosynthetic networks can be creatively redesigned to enable de novo production of diverse vitamin E compounds, rather than simply replicating or incrementally optimizing native plant metabolic pathways. Equally important is how advanced fermentation strategies can be integrated to achieve scalable, high‐efficiency, and cost‐effective production.

## FUTURE PERSPECTIVES

Research on vitamin E is increasingly shifting from descriptive biology toward precision regulation and efficient application. Future priorities include elucidating its fine‐scale regulatory mechanisms governing its biosynthesis, developing high‐yield biotechnological platforms, and expanding its functional scopes in nutrition and industrial applications.

### Resolving enzymatic bottlenecks and intelligent engineering

The efficiency of vitamin E production in microbial and plant‐based systems is fundamentally constrained by precursor availability and enzymatic catalytic performance. Future research should therefore focus on resolving several key rate‐limiting steps. Precursor supply (upstream bottlenecks) remains a major constraint, as the limited flux of HGA from the shikimate pathway and PDP or GGPP from the MEP/MVA pathways restricts overall pathway throughput (Valentin et al., 2006; Vom Dorp et al., 2015; Gutbrod et al., 2019). In addition, competitive diversion of intermediates toward chlorophyll and carotenoid biosynthesis further reduces precursor availability for vitamin E production.

At the pathway entry point, HPT/VTE2 represents a primary limiting factor for total tocopherol yield, while HGGT activity largely determines the yield and compositional purity of high‐value tocotrienols in seeds. Downstream, VTE3 and VTE4, encoding MPBQ methyltransferase and γ‐TMT, respectively, function as key regulators of isomer composition (Shintani and Dellapenna, 1998; Bergmüller et al., 2003; Cheng et al., 2003; Van Eenennaam et al., 2003). Insufficient activity of these enzymes results in incomplete conversion to the biologically most active α‐forms. Addressing these interconnected bottlenecks will require a systematic and integrated engineering strategy that combines pathway flux optimization with targeted enhancement of enzymatic specificity and efficiency.

#### Upstream metabolic flux optimization

Future research should be directed toward enhancing flux through the shikimate and MEP/MVA pathways. This can be achieved by overexpressing key regulatory enzymes, such as HPPD, GGDR, and GGPP synthase, disrupting negative regulatory genes (e.g., *HGO*), or introducing feedback‐insensitive enzyme variants. Together, these approaches are expected to increase the accumulation of HGA and isoprenoid precursors, thereby alleviating upstream supply limitations that constrain vitamin E biosynthesis.

#### Substrate channeling via enzyme fusion

In both microbial and plant systems, the use of optimized linker peptides to fuse key enzymes (e.g., SyHPT and AtVTE1) can establish artificial substrate channeling (Shen et al., 2020). This strategy facilitates the direct transfer of intermediates between catalytic sites, thereby increasing reaction efficiency and reducing metabolic losses. By minimizing the diffusion of unstable or potentially toxic intermediates, such as MGGBQ (Oram et al., 2001; Gruszecki and Strzałka, 2005), substrate channeling minimizes losses due to oxidation or diversion into competing pathways. This strategy has shown considerable promise in complex multistep biosynthetic cascades (Wissner et al., 2025) and represents a compelling paradigm for the rational design of efficient, multi‑enzyme biocatalytic systems.

#### Intelligent protein evolution

The convergence of molecular dynamics (MD) simulations and machine learning (ML) has established a powerful paradigm for the precise engineering of enzyme activity and stability. Recent advances in artificial intelligence, particularly the development of protein language models such as ESM‐2 (Lin et al., 2023) and ProteinBERT (Brandes et al., 2022), have greatly expanded the capabilities of computational protein design. Trained on a large‐scale sequence database, these models capture evolutionary constraints and structural semantics, enabling the construction of robust sequence‐function relationships. Building on this foundation, AI‐driven models can systematically predict catalytic efficiency and thermal stability across enzyme variants (Cheng et al., 2024), while MD simulations provide dynamic, atomistic insights that support the virtual screening of promising candidates (Mapar et al., 2024; Uba et al., 2024). Together, this integrated *in silico* strategy has the potential to significantly accelerate the discovery and optimization of high‐performance enzymes.

To translate computational predictions into functional solutions, intelligent design strategies must be coupled with experimental high‐throughput screening (HTS). Assays based on fluorescence, colorimetric readouts, or growth‐coupled selection system enable rapid functional evaluation of large mutant libraries, facilitating efficient identification of optimized enzyme variants.

### Plant systems: Precision biofortification and stress resilience

Future research in plant biofortification should prioritize the coordinated optimization of subcellular transport and systemic regulatory networks to alleviate persistent bottlenecks in vitamin E biosynthesis. Such an integrated perspective will be essential for improving biosynthetic efficiency beyond single‐gene or pathway‐level interventions.

#### Ideal carrier plants for vitamin E biofortification

Plant species differ substantially in both total vitamin E content and homolog composition, providing a broad genetic resource for rational biofortification. Ideal carrier crops should combine high oil content, efficient synthesis of precursors such as HGA, PDP, and GGPP, minimal competition from parallel metabolic pathways, and strong amenability to genetic transformability. Priority should be given to species that combine high oil content with efficient vitamin E precursor synthesis capabilities, such as oil palm, barley, and certain microalgae (Jayusman et al., 2014; Zeng et al., 2020). Oil content is closely linked to vitamin E (tocopherol) accumulation, as tocopherols are lipophilic molecules whose synthesis, sequestration, and storage are tightly associated with lipid metabolism (Chu et al., 2023). Elevated oil content not only provides a natural storage medium for tocopherols but also often reflects a stronger supply of shared precursors, thereby supporting higher vitamin E yields. Accordingly, future crop optimization may focus on species with distinctive tocopherol profiles or exceptionally high oil content, which can be engineered as platforms for the targeted production of high‑value vitamin E homologs, enhancing both nutritional impact and economic return.

#### Elucidating transmembrane transport mechanisms

While the absorption and transport of α‑tocopherol in humans have been relatively well characterized, vitamin E biosynthesis in plants spans multiple cellular compartments. Future studies should therefore adopt “transportomics” approaches to identify and characterize membrane transporters for the movement of key precursors, such as homogentisic acid, phytol, and isopentenyl diphosphate (Krumpochova et al., 2012). Targeted manipulation of these transporters could enable more precise allocation of metabolic intermediates, thereby enhancing spatial coordination and overall efficiency of vitamin E biosynthesis.

#### Constructing an integrated regulatory framework

Moving beyond simple gene overexpression, future research should integrate chromatin immunoprecipitation sequencing (ChIP‑seq), Y1H assays, and CRISPR‑Cas9‐based approaches to systematically validate interactions between transcription factors and promoters of vitamin E biosynthetic genes. Elucidating how these transcriptional modules integrate environmental and endogenous signals, such as light, hormones, and oxidative stress, will be essential for building a coherent regulatory network governing vitamin E biosynthesis (Kruk et al., 2005; Ouyang et al., 2011; Ren et al., 2011; Jiang et al., 2017; Bao et al., 2020).

### Microbial systems: Next‐generation cell factories

Microbial cell factories represent a promising platform for the sustainable and high‐purity production of vitamin E.

#### Selection of advanced chassis strains

Although *S. cerevisiae* and *E. coli* are commonly employed as engineering hosts, *Y. lipolytica* offers distinct advantages due to its abundant acetyl‐CoA supply and native lipid bodies, which provide a favorable intracellular environment for the accumulation and storage of hydrophobic tocopherols (Wang et al., 2024a; Xiang et al., 2025).

#### Progress and challenges in microbial tocopherol synthesis

Although microbial synthesis of tocotrienols has been successfully demonstrated, fermentative production of tocopherols remains relatively underexplored. One critical bottleneck is the limited availability of PDP. Functional expression of GGDR within engineered vitamin E biosynthetic pathways could be pivotal, as this enzyme catalyzes the conversion of GGPP to PDP, a prerequisite for tocopherol biosynthesis.

#### Secretion engineering and tolerance mechanisms

Intracellular accumulation of target compounds frequently results in cytotoxicity, which not only impairs cell growth and metabolic activity but may also exert feedback inhibition on the biosynthetic pathway, ultimately limiting product titers. Moreover, downstream recovery based on cell disruption is energy‐intensive and increases process complexity. Systematic studies on secretory production have revealed that redirecting efforts toward screening and engineering endogenous ABC transporters to promote active efflux represents an effective strategy to mitigate intracellular toxicity. This approach enables *in situ* product separation, simplifies downstream purification, and improves overall process economics (Jiao et al., 2022, 2023).

#### Integrated bio‐chem hybrid approaches

The integration of microbial farnesene fermentation with streamlined chemical catalysis has already demonstrated transformative potential (Zhu et al., 2014; Ye et al., 2022). Further development of stereoselective catalytic methods in downstream chemical steps could enable the large‐scale production of single‐isomer, natural‐form vitamin E (Takaya et al., 1987; Schmid et al., 1996; Wang et al., 2008; Woodmansee et al., 2010; Bonrath et al., 2021).

### Non‐conventional sources and rare homologs

Emerging research into non‐traditional sources and rare vitamin E homologs is broadening the scope of the field. In microalgae, nitrogen depletion selectively enhances α‐tocopherol accumulation without affecting tocomonoenols or tocotrienols, indicating alternative biosynthetic branches that could be exploited for targeted production (Montoya‐Arroyo et al., 2024). Studies of rare homologs, such as α‐T1, demonstrate cellular uptake and metabolic behavior in HepG2 cells comparable to that of α‐tocopherol, accompanied by excellent biosafety and an absence of cytotoxicity (Montoya‐Arroyo et al., 2021). These underexplored compounds may possess distinct physiological and therapeutic potential, meriting further research on their bioavailability, antioxidant capacity, and health effects. Deciphering these rare homologs will deepen our understanding of the vitamin E family and reveal new opportunities for functional applications.

## CONCLUSION

A century after its discovery, vitamin E research has evolved from a nutritional curiosity to a multidisciplinary frontier encompassing plant biochemistry, redox biology, and metabolic engineering. Once regarded primarily as a lipid‐soluble antioxidant, vitamin E is now recognized as a dynamic regulator of cellular signaling, stress adaptation, and metabolic balance. The convergence of genomics, synthetic biology, and intelligent enzyme design is redefining how this essential molecule can be produced, optimized, and applied across biological systems. Future advances will depend on integrative approaches that link biosynthetic regulation, transport dynamics, and molecular evolution with scalable industrial innovation. By harnessing natural diversity, rational design, and sustainable production, researchers can transform vitamin E from a classical antioxidant into a model compound for precision metabolic engineering. In the coming years, the pursuit of vitamin E research will continue to illuminate the intricate dialogue between structure and function, biology and technology, reaffirming its central role in both plant metabolism and human health.

## CONFLICTS OF INTEREST

The authors declare no conflicts of interest.

## AUTHOR CONTRIBUTIONS

R.Z., L.W., and L.Z. conceived the study. R.Z., Y.R., Y.Z, H.Z., Y.Z.L., and Y.L. collected the literature. R.Z. drafted the manuscript. R.Z. prepared all the figures. L.Z. and L.W. critically revised and edited the manuscript. All authors read and approved the final version of the manuscript.
